# Association of Nationwide Hepatitis B Vaccination and Antiviral Therapy Programs With End-Stage Liver Disease Burden in Taiwan

**DOI:** 10.1001/jamanetworkopen.2022.22367

**Published:** 2022-07-18

**Authors:** Chun-Ju Chiang, Jing-Rong Jhuang, Ya-Wen Yang, Bo-Zhi Zhuang, San-Lin You, Wen-Chung Lee, Chien-Jen Chen

**Affiliations:** 1 Institute of Epidemiology and Preventive Medicine, College of Public Health, National Taiwan University, Taipei, Taiwan; 2 Taiwan Cancer Registry, Taipei, Taiwan; 3 College of Medicine and Big Data Research Centre, Fu-Jen Catholic University, New Taipei, Taiwan; 4 Genomics Research Center, Academia Sinica, Taipei, Taiwan; 5 College of Medicine, Fu-Jen Catholic University, New Taipei, Taiwan

## Abstract

**Importance:**

Chronic infections of hepatitis B and C viruses are the main causes of end-stage liver disease (ESLD) worldwide. The successful control of viral hepatitis is critical to reducing the burden of ESLD.

**Objective:**

To examine the association of 2 world-first nationwide neonatal hepatitis B vaccinations and national antiviral therapy programs implemented in Taiwan (in 1984 and 2003, respectively) with the burden of ESLD.

**Design, Setting, and Participants:**

This cohort study included children and adults aged 5 to 39 years from the National Cancer Registry Database and the National Death Registry Database between 1979 and 2018 in Taiwan. Individuals who died from chronic liver disease (CLD) and cirrhosis and hepatocellular carcinoma (HCC) or were diagnosed with HCC were included for analysis. Analyses were performed in January 2021.

**Main Outcomes and Measures:**

The main outcomes were measures of age, period, and cohort associations with CLD mortality and HCC incidence and mortality using an age-period-cohort analysis.

**Results:**

Among the 43 604 individuals (mean [SD] age, 33.3 [6.0] years; 37 755 men [86.6%]) with ESLD in the cohort, there were 17 904 CLD deaths, 11 504 HCC deaths, and 14 196 HCC incident events. There was a significant increase in all 3 disease burdens after age 20 years. From 2004 to 2018, CLD mortality decreased by 26% (adjusted rate ratio [aRR], 0.74; 95% CI, 0.70-0.82), HCC mortality decreased by 50% (aRR, 0.50; 95% CI, 0.45-0.58), and HCC incidence decreased by 53% (aRR, 0.47; 95% CI, 0.44-0.52). A sharply declining association by cohort was observed from the 1982 to 1986 birth cohort to the 2007 to 2011 birth cohort, during which CLD mortality decreased by 82% (aRR, 0.18; 95% CI, 0.00-1.13), HCC mortality decreased by 63% (aRR, 0.37; 95% CI, 0.09-0.68), and HCC incidence decreased by 80% (aRR, 0.20; 95% CI, 0.00-0.48). The associations of age, period, and cohort were similar between male and female individuals and between urban and rural areas. The percentage of chronic hepatitis B infections in patients with HCC decreased from 83.3% (95% CI, 79.7%-86.5%) for those born from 1980 to 1984 to 55.6% (95% CI, 21.2%-86.3%) (from 2000 to 2004).

**Conclusions and Relevance:**

These findings suggest that the national hepatitis B vaccination program and the antiviral therapy program jointly were associated with substantial reductions in the burden of ESLD in Taiwan.

## Key Points

**Question:**

What is the association of nationwide hepatitis B vaccination and antiviral therapy programs with the burden of end-stage liver disease (ESLD) in Taiwan?

**Findings:**

In this cohort study of children and adults aged 5 to 39 years, 43 604 individuals with ESLD were noted, and significant decreases were observed in chronic liver disease (CLD) mortality, hepatocellular carcinoma (HCC) mortality, and HCC incidence in the period of 2004 to 2018 in Taiwan. Cohort analysis also found a sharply declining trend in CLD mortality, HCC mortality, and HCC incidence from the 1982 to 1986 birth cohort to the 2007 to 2011 birth cohort.

**Meaning:**

The findings of this study suggest that the nationwide hepatitis B vaccination and antiviral therapy programs jointly were associated with substantial reductions in ESLD burden in Taiwan.

## Teaser

This cohort study examines the associations of nationwide hepatitis B vaccination and antiviral therapy programs implemented in Taiwan with the burden of end-stage liver disease by age, period, and cohort.

## Introduction

End-stage liver disease (ESLD), including chronic liver diseases (CLDs) and cirrhosis and liver cancer, account for approximately 1 in 50 deaths worldwide.^1,2^ Liver cancer is the sixth most commonly diagnosed cancer and the third leading cause of cancer death globally.^3^ Chronic infections of hepatitis B virus (HBV) and hepatitis C virus (HCV) are major causes of ESLD in most parts of the world.^4^ HBV infection is prevalent in Africa and the Western Pacific region, and HCV infection is prevalent in European and Eastern Mediterranean areas.^1^ The successful control of viral hepatitis is critical in reducing the global burden of ESLD.

In Taiwan, liver cancer, primarily hepatocellular carcinoma (HCC), became the most common cancer and the leading cause of cancer death in the 1980s.^5,6^ Nearly 80% of patients with CLD and HCC were seropositive for the HBV surface antigen (HBsAg).^7,8^ Mother-to-infant transmission is a major route of HBV transmission^9,10^; once infected early in life, the chronic carrier rate and the risk of ESLD later in life increase substantially. The first nationwide neonatal HBV vaccination program worldwide was implemented in Taiwan in July 1984.^11^ Serological surveys found a significant decrease in chronic HBV infections from 10% in unvaccinated birth cohorts to 0.5% in vaccinated ones.^12,13,14^ Moreover, HCC, CLD, and infant fulminant hepatitis have been markedly reduced in vaccinated birth cohorts.^15,16,17^ In 1992, the World Health Organization (WHO) recommended incorporating the HBV vaccine into national immunization programs. By the end of 2019, universal infant HBV vaccination programs had been adopted in 189 countries.^1,18^

Treatment for chronic HBV infection began in 1985 and has been progressively improved in many countries. The main goal of antiviral therapy in patients with chronic hepatitis B is to suppress HBV replication, delay progression to cirrhosis, and reduce the risks of HCC and liver-related deaths.^19^ The first nationwide antiviral therapy program worldwide was implemented in Taiwan in October 2003. The burden of ESLD in Taiwan has declined since the launch of this program.^17,20,21,22^ Studies have projected that the ESLD burden in Taiwan will continue to decline until 2035.^23,24^ The WHO has recommended nationwide antiviral therapy programs for the global control of HBV infection since 2015.^1^

The influence of the HBV vaccination program on the burden of ESLD is likely to manifest as a cohort association. In contrast, the influence of the antiviral therapy program is expected to display as a period association. In Taiwan, birth cohorts comprising individuals who were vaccinated at the launch of the vaccination program have now reached up to age 35 years. Most of these individuals have also benefited from the antiviral therapy program at various times in their life course. To evaluate the joint association of the HBV vaccination and antiviral therapy programs with the burden of ESLD, we must account for the combined variables of age, period, and cohort. Age-period-cohort analysis has been widely used to determine associations of age, period, and cohort with disease incidence and mortality.^25,26,27,28^ This study conducted an age-period-cohort analysis of CLD mortality, HCC mortality, and HCC incidence between 1979 and 2018 in Taiwan.

## Methods

This cohort study was approved by the National Taiwan University Research Ethics Committee with a waiver of informed consent owing to the lack of personal information and use of secondary data in the study. The Strengthening the Reporting of Observational Studies in Epidemiology (STROBE) reporting guideline for observational studies was used in the revision of this article.

### Study Cohorts

This cohort study included individuals with incident HCC aged 5 to 39 years from the Taiwan Cancer Registry Database and CLD or HCC deaths among individuals aged 5 to 39 years from the National Death Registry Database between 1979 and 2018. The Taiwan Cancer Registry is a national population-based cancer registry system with comprehensive, high-quality data.^29,30,31^ Because all deaths must be registered by law within 1 month of occurrence with the Department of Statistics of the Ministry of Health and Welfare, the computerized death registry database in Taiwan is complete and accurate. National population numbers were derived from the Department of Statistics of the Ministry of the Interior.

The individuals were classified as having HCC (*International Classification of Diseases for Oncology, Third Edition* [*ICD-O-3*]^32^ topography code: 1550 or C220, with morphology codes 8170-8175), mortality from CLD (*International Classification of Diseases, Ninth Revision* [*ICD-9*]^33^ code 571 and *International Statistical Classification of Diseases and Related Health Problems, Tenth Revision* [*ICD-10*]^34^ code K70/K73-74), or mortality from HCC (*ICD-9* code 1550/1552 and *ICD-10* code C220/C229). The individuals were grouped by age at diagnosis or death into 7 age groups, each spanning 5 years (ages 5-9, 10-14, 15-19, 20-24, 25-29, 30-34, and 35-39 years). We excluded individuals younger than 5 years to exclude patients with hepatoblastoma. The individuals were grouped by year of diagnosis or death, each spanning 5 years (1979-1983, 1984-1988, 1989-1993, 1994-1998, 1999-2003, 2004-2008, 2009-2013, and 2014-2018). A total of 56 cells were derived from age-by-period cross-classification. We calculated the year of birth for each cell by subtracting the age at diagnosis or death from the year of diagnosis or death. Fourteen 5-year birth cohorts were defined (1942-1946, 1947-1951, …, 2007-2011). Birth cohorts of the individuals in 2 adjacent birth cohort groups overlapped, but this was ignored here for simplicity.

### Nationwide HBV Vaccination and Antiviral Therapy Programs in Taiwan

In 1984, the world’s first nationwide HBV vaccination program was launched in Taiwan.^11^ During the first 2 years of the program, only newborns of mothers with high risk who were seropositive for HBsAg were vaccinated. After 1986, the program was extended to all newborns. Preschool children who were not vaccinated against HBV in infancy were vaccinated in 1987 to 1989, and primary school children and adolescents were vaccinated on a fee-for-service basis in 1988 to 1990. Since 1992, recombinant HBV vaccines have replaced plasma-derived vaccines and have been given in 3 doses at birth and ages 1 and 6 months. By 2019, the 3-dose coverage rate for infants was 98.4% in Taiwan.^35^

In 2003, the world’s first national antiviral therapy program for chronic hepatitis B and C was implemented in Taiwan.^17,20^ Since then, all patients with high risk who develop cirrhosis or HCC have been reimbursed for antiviral therapy. After 2009, patients with moderate to high risk of developing ESLD were reimbursed for more potent antiviral agents, including entecavir and tenofovir. Direct-acting antiviral agents for chronic hepatitis C have also been reimbursed since 2017.^36^

### Statistical Analysis

Incidence and mortality rates (per 100 000 person-years) were standardized to the WHO 2000 Standard Population^37^ proportions (truncated age interval 5 to 39 years). For the age-period-cohort model, we used a Poisson regression model to analyze the data of the 56 cells from the age-by-period cross-classification table. A total of 7 models were evaluated, including 3 one-factor models (the respective age, period, and cohort models), 3 two-factor models (the age-period, age-cohort, and period-cohort models), and 1 three-factor age-period-cohort model. To avoid the problem of nonidentifiability encountered in the full-fledged age-period-cohort model, the constant-relative-variation constraint^27^ was used to estimate the age, period, and cohort associations. This method has successfully demonstrated the association between birth cohort trends of oral cancer incidence rates and betel-nut consumption in Taiwan.^28^ We compared the goodness-of-fit of the 7 models using deviance statistics and the likelihood ratio tests.^25,26^ We selected the best model and used it to fit the incidence and mortality data. Adjusted rate ratios (aRRs) and 95% CIs were estimated from the age-period-cohort model. Two-sided statistical significance was set at *P* < .05. All statistical analyses were performed using SAS statistical software version 9.4 (SAS Institute).

## Results

**Figure 1.**
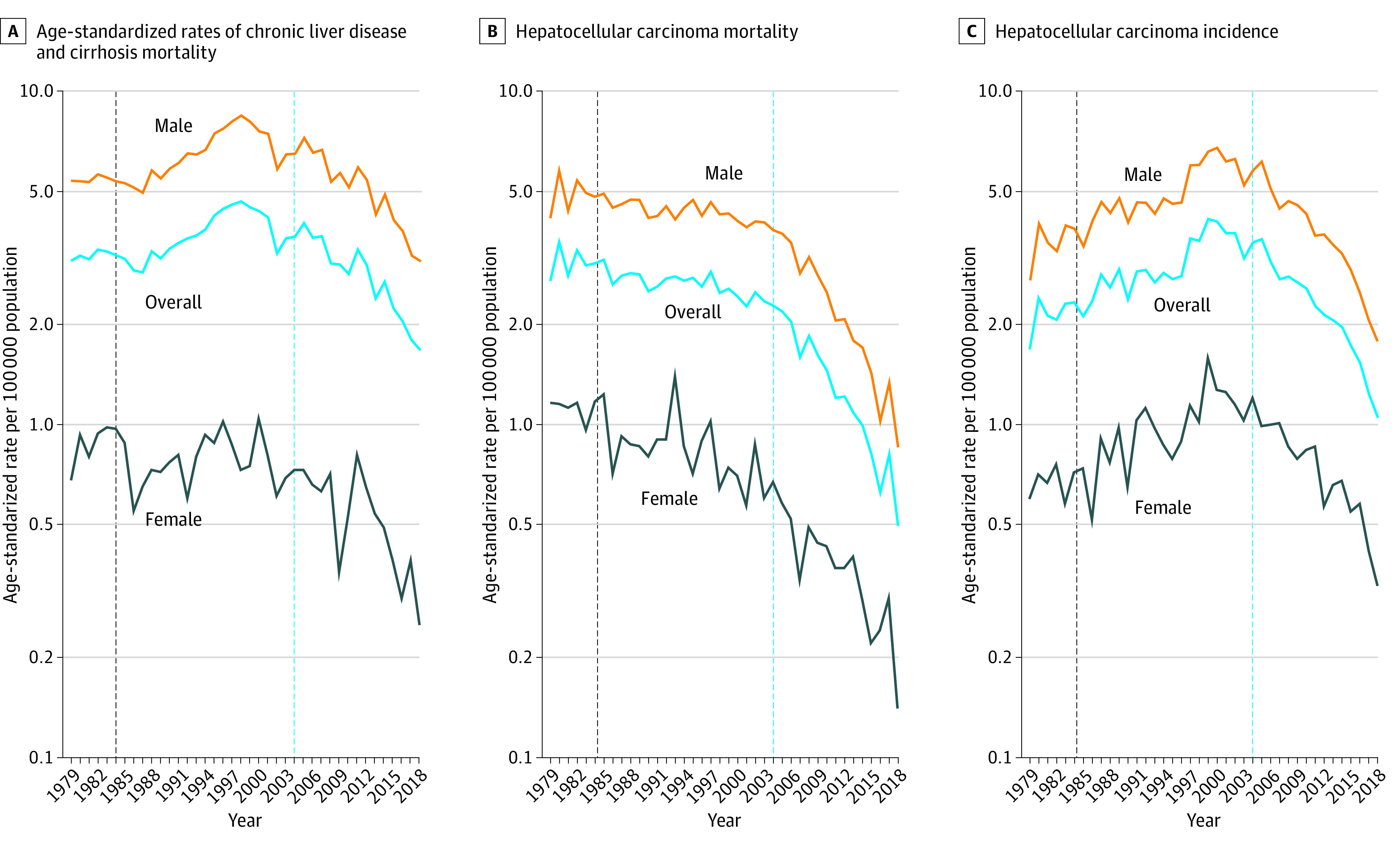
Age-Standardized Rates of Chronic Liver Disease and Cirrhosis Mortality, Hepatocellular Carcinoma Mortality, and Hepatocellular Carcinoma Incidence From 1979 to 2018 in Taiwan The age-standardized rates were truncated by age interval of 5 to 39 years and adjusted with the World Health Organization 2000 Standard Population.^37^ The black dotted line marks the implementation of the hepatitis B vaccination program, and the blue dotted line marks the initiation of the antiviral therapy program.

Among the 43 604 individuals (mean [SD] age, 33.3 [6.0] years; 37 755 men [86.6%]) with ESLD in the cohort, there were 17 904 CLD deaths (16 073 [89.8%] among male individuals; 1831 [10.2%] among female individuals), 11 504 HCC deaths (9716 [84.5%] among male individuals; 1788 [15.5%] among female individuals), and 14 196 HCC incident cases (11 966 [84.3%] among male individuals; 2230 [15.7%] among female individuals) aged 5 to 39 years between 1979 and 2018 in Taiwan. Figure 1 displays trends in the age-standardized CLD mortality, HCC mortality, and HCC incidence per 100 000 population from 1979 to 2018 in Taiwan for overall and stratified by sex. CLD mortality increased before 1999 and then decreased. HCC mortality decreased gradually before 2004 but has decreased rapidly since then. HCC incidence increased before 2001 and then decreased.

**Figure 2.**
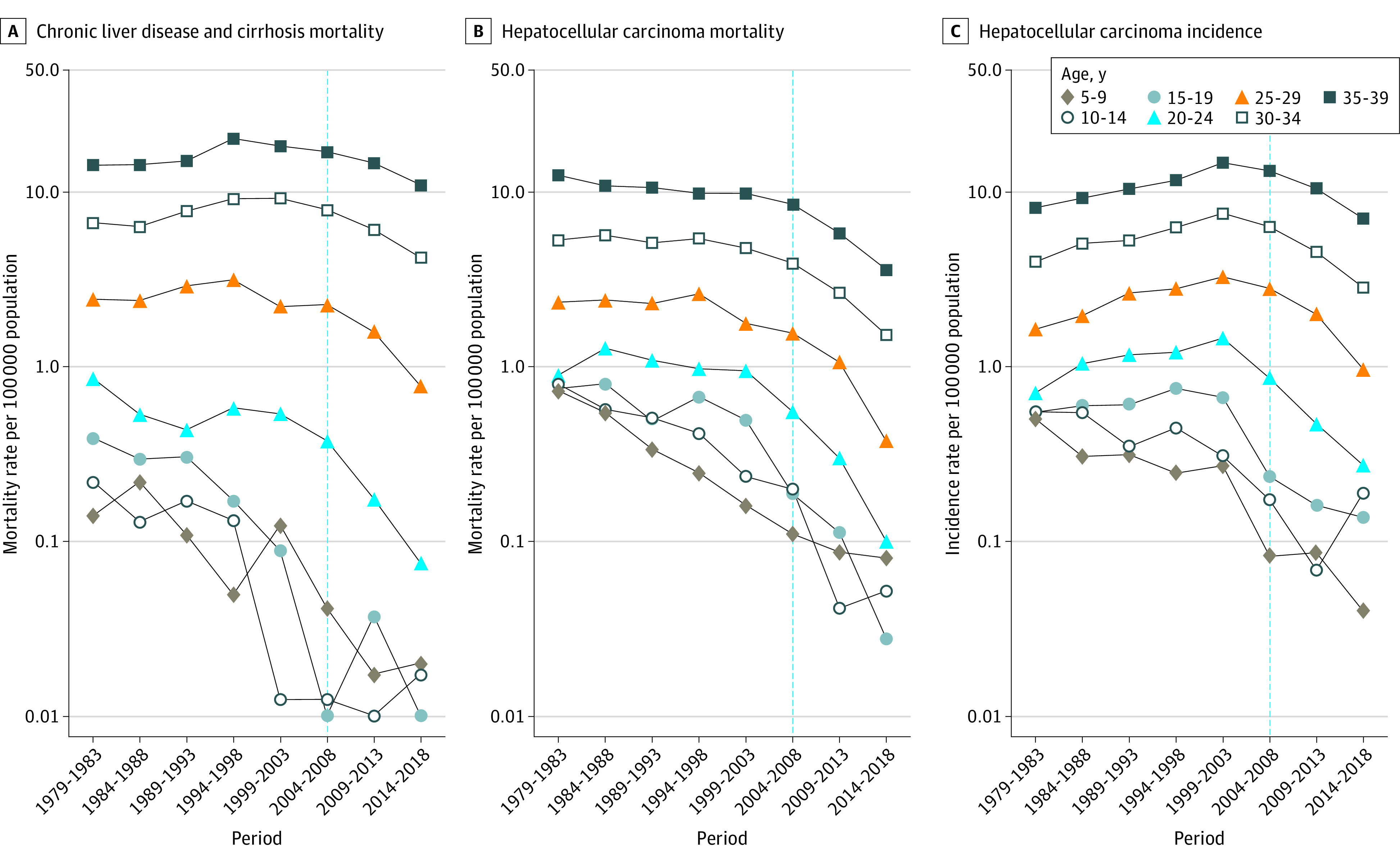
Age-Specific Rates of Chronic Liver Disease and Cirrhosis Mortality, Hepatocellular Carcinoma Mortality, and Hepatocellular Carcinoma Incidence From 1979 to 2018 in Taiwan The blue dotted line marks the initiation of the antiviral therapy program.

Figure 2 displays the period trends in age-specific rates overall in Taiwan. The period trends before 2004 increased (for CLD mortality and HCC incidence) or leveled off (for HCC mortality) in individuals aged 20 to 39 years but decreased (for all 3 disease burdens) in those aged 5 to 19 years. After 2004, the period trend for all 3 disease burdens declined consistently for all ages. The findings were similar for males and females (eFigure 1 and eFigure 2 in the Supplement) and in urban (metropolis and cities) and rural (towns and villages) areas (eFigure 3 and eFigure 4 in the Supplement).

**Figure 3.**
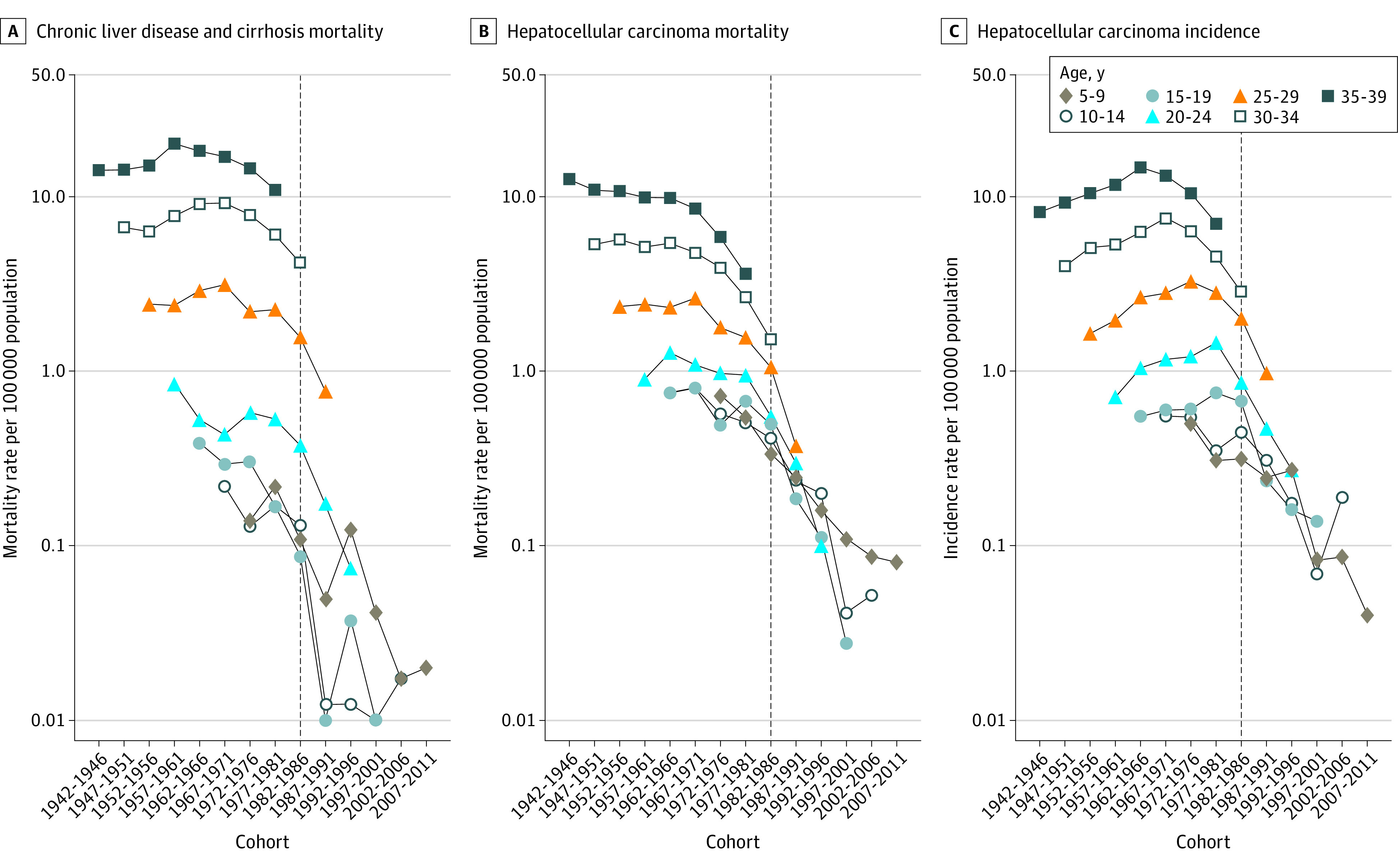
Age-Specific Rates of Chronic Liver Disease and Cirrhosis Mortality, Hepatocellular Carcinoma Mortality, and Hepatocellular Carcinoma Incidence From 1979 to 2018 in Taiwan The black dotted line marks the implementation of the hepatitis B vaccination program.

Figure 3 displays the cohort trends in age-specific rates overall in Taiwan. The cohort trends before 1967 increased (for CLD mortality and HCC incidence) or decreased slightly (for HCC mortality). The cohort trends for all 3 disease burdens showed a gradual decline for those born after 1971 and a sharp decline for those born after 1986. The findings were similar for male and female individuals (eFigure 5 and eFigure 6 in the Supplement) and in urban and rural areas (eFigure 7 and eFigure 8 in the Supplement).

**Figure 4.**
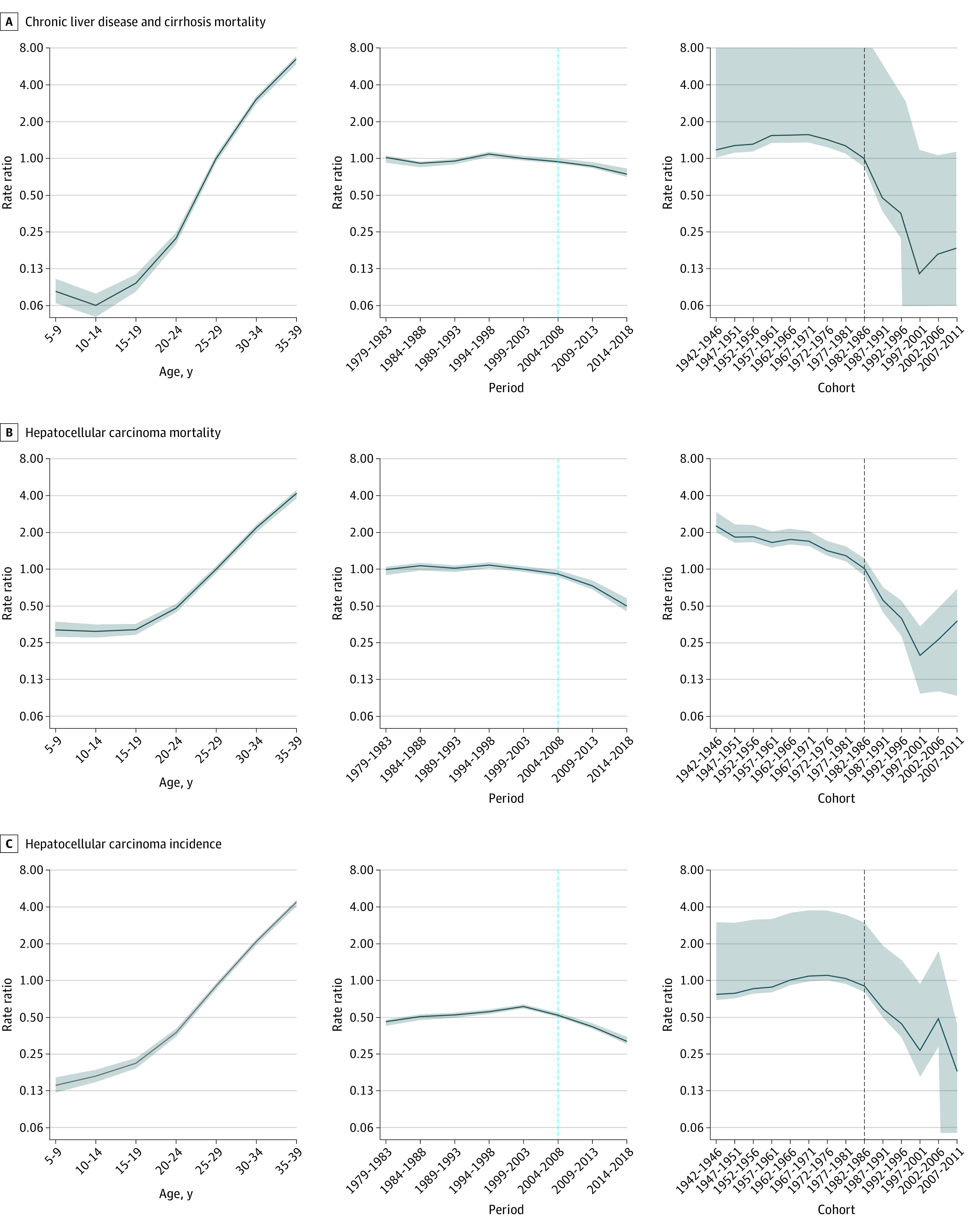
Associations of Age, Period, and Cohort With Chronic Liver Disease and Cirrhosis Mortality, Hepatocellular Carcinoma Mortality, and Hepatocellular Carcinoma Incidence From 1979 to 2018 in Taiwan The black dotted line marks the implementation of the hepatitis B vaccination program, and the blue dotted line marks the initiation of the antiviral therapy program. Shaded areas indicate 95% bootstrapped CIs.

eTable 1 in the Supplement shows that all 3 temporal analyses (age, period, and cohort) were statistically significant. Figure 4 displays the associations of age, period, and cohort estimated by the age-period-cohort model overall. There was more variability in the estimates for ages younger than 20 years, judging from the wider 95% CIs. After that, all 3 disease burdens increased with age. The association with age was more conspicuous for CLD mortality (105-fold greater for individuals aged 35-39 years compared with their younger counterparts) than for HCC mortality (15-fold greater) and HCC incidence (30-fold greater).

For the association with period, CLD and HCC mortalities began to decline after 1999 (Figure 4). After 2004, all 3 disease burdens exhibited a consistent downward trend, with CLD mortality reduced by 26% (aRR, 0.74; 95% CI, 0.70-0.82), HCC mortality reduced by 50% (aRR, 0.50; 95% CI, 0.45-0.58), and HCC incidence reduced by 53% (aRR, 0.47; 95% CI, 0.44-0.52) (Table).

**Table.** Change in CLD and Cirrhosis Mortality, HCC Mortality, and HCC Incidence Over Periods and Cohorts in Taiwan

Assessment	CLD and cirrhosis mortality			HCC mortality			HCC incidence		
	expβ (95% CI)^a^	aRR (95% CI)	Change, %	expβ (95% CI)^a^	aRR (95% CI)	Change, %	expβ (95% CI)^a^	aRR (95% CI)	Change, %
Period									
1999-2003	1.07 (1.03-1.13)	1 [Reference]	0 [Reference]	1.13 (1.07-1.19)	1 [Reference]	0 [Reference]	1.32 (1.27-1.39)	1 [Reference]	0 [Reference]
2014-2018	0.79 (0.75-0.89)	0.74 (0.70-0.82)	−26	0.57 (0.51-0.65)	0.50 (0.45-0.58)	−50	0.62 (0.58-0.68)	0.47 (0.44-0.52)	−53
Prevaccination cohort									
1967-1971	2.22 (1.92-5.79)	1 [Reference]	0 [Reference]	1.84 (1.68-2.23)	1 [Reference]	0 [Reference]	1.63 (1.48-5.64)	1 [Reference]	0 [Reference]
1982-1986	1.42 (1.21-5.96)	0.64 (0.05-0.74)	−36	1.11 (0.96-1.34)	0.60 (0.49-0.65)	−40	1.36 (1.21-4.48)	0.83 (0.24-0.92)	−17
Postvaccination cohort									
1982-1986	1.42 (1.21-5.96)	1 [Reference]	0 [Reference]	1.11 (0.96-1.34)	1 [Reference]	0 [Reference]	1.36 (1.21-4.48)	1 [Reference]	0 [Reference]
2007-2011	0.26 (0.00-1.60)	0.18 (0.00-1.13)	−82	0.41 (0.10-0.76)	0.37 (0.09-0.68)	−63	0.28 (0.00-0.66)	0.20 (0.00-0.48)	−80

Abbreviations: aRR, adjusted rate ratio; CLD, chronic liver disease; expβ, exponentiated β; HCC, hepatocellular carcinoma.

^a^ Effect size from the age-period-cohort model.

For cohorts born before 1971, CLD mortality and HCC incidence increased, but HCC mortality decreased (Figure 4). For cohorts born after 1971, all 3 disease burdens decreased gradually, and for those born after 1986, sharply. CLD mortality decreased by 36% (aRR, 0.64; 95% CI 0.05-0.74), HCC mortality decreased by 40% (aRR, 0.60; 95% CI, 0.49-0.65), and HCC incidence decreased by 17% (aRR, 0.83; 95% CI, 0.24-0.92) from the 1967 to 1971 birth cohort to the 1982 to 1986 birth cohort. From the 1982 to 1986 birth cohort to the 2007 to 2011 birth cohort, reductions were sharper, with CLD mortality decreasing by 82% (aRR, 0.18; 95% CI, 0.00-1.13), HCC mortality decreasing by 63% (aRR, 0.37; 95% CI, 0.09-0.68), and HCC incidence decreasing by 80% (aRR, 0.20; 95% CI, 0.00-0.48) (Table). There was an increase in risk in the most recent cohorts, possibly due to random variation, judging from the wide 95% CIs.

The findings from the age-period-cohort analysis were similar for male and female individuals (eTable1, eFigure 9, and eFigure 10 in the Supplement) and in urban and rural areas (eTable 2, eFigure 11, and eFigure 12 in the Supplement). The percentage of chronic HBV infections in individuals with HCC decreased from 83.3% (95% CI, 79.7%-86.5%) for those born from 1980 to 1984 to 55.6% (95% CI, 21.2%-86.3%) for those born from 2000 to 2004 (eTable 3 in the Supplement).

## Discussion

In this cohort study using empirical data, we performed an age-period-cohort analysis of CLD mortality, HCC mortality, and HCC incidence from 1979 to 2018 in Taiwan to assess the association of national programs of HBV vaccination and antiviral therapy in reducing the ESLD burden. The cohort analysis indicated a significant reduction in ESLD burden (63% to 82%) for cohorts born after the national HBV vaccination program was implemented. Likewise, the period analysis indicated a significant reduction in ESLD burden (26% to 53%) after the national antiviral therapy program. In several sensitivity analyses, similar results were found for male and female individuals and in urban and rural areas. Our findings indicate that the combination of the HBV vaccination and antiviral therapy programs was associated with significantly reducing the burden of ELSD in Taiwan, regardless of sex or region.

A study by Chang et al^38^ examined the secular trend of the incidence and mortality of HCC in Taiwan using the age-period-cohort model. They found that the HBV vaccination program reduced the incidence of HCC in boys but not girls. However, their study did not exclude patients younger than 5 years who were mainly diagnosed with hepatoblastoma unrelated to a viral infection. The shorter period (from 1980 to 2009) of their study resulted in the underestimation of the cohort association of the HBV vaccination program and an inability to detect the period association of the antiviral therapy program. A 2021 study^17^ assessing the association of serial preventive strategies for HCC occurrence and deaths in Taiwan did not examine associations by age, period, and cohort using the unified framework of the age-period-cohort model. A plot of age-standardized rates over time can sometimes mislead without an age-period-cohort model to disentangle the associations of age, period, and cohorts. For example, from Figure 1 of our study, one gets an impression that CLD mortality and HCC incidence increased (as opposed to decreased) after implementing the HBV vaccination program in 1984.

The finding that the percentage of chronic HBV infections in individuals with HCC decreased for those born after 1984 further suggests that the hepatitis B vaccination program was associated with markedly reduced incidence of HBV-related HCC in Taiwan. Furthermore, primary school children and adolescents were vaccinated on a fee-for-service basis between 1988 and 1990, suggesting that some children and adolescents born between 1970 and 1983 may have been vaccinated with catch-up doses. This indicates that the HBV vaccination program may also influence those cohorts born several years before 1984. Studies have reported that the prevalence of HBsAg began to decline 5 years before 1984 in Taiwan.^39,40^ Routine screening of HBsAg in Taiwan’s blood banks started in 1974, and disposable needles and syringes were widely promoted in 1980. These preventive strategies may have conferred a protective association for the unvaccinated cohorts.^40^ This study also found that the ESLD burden in Taiwan had already declined in birth cohorts from 1967 to 1971 to 1982 to 1986.

Our findings also indicate a dramatic decline in CLD mortality, HCC mortality, and HCC incidence after 2004 associated with the national chronic hepatitis antiviral therapy program. However, CLD and HCC mortalities declined approximately 5 years before 2004. The National Health Insurance (NHI) Program was implemented for all citizens in Taiwan in 1995 and has provided widespread access to the diagnosis and treatment of liver diseases.^41^ The NHI program may have contributed to the earlier reductions in CLD and HCC mortality rates.

Taiwan has high-quality nationwide health registry databases.^42^ The availability of long-term, high-quality cancer registry and death certificate databases at the national level is crucial for evaluating various prevention programs to eliminate viral hepatitis and HCC. In Taiwan, the population-attributable risk of developing HCC was much higher for HBV than HCV infections.^43,44^ Our study corroborates successful HCC prevention and control through nationwide HBV vaccination and antiviral treatment programs. The HBV vaccination coverage rates in Taiwan increased from 88.8% to 98.4% for birth cohorts from 1984 to 2019.^35^ The Taiwanese government began fully subsidizing the antiviral treatment of chronic hepatitis B and C in 2003 and antiviral prophylaxes for pregnant mothers with high viral titers in 2018 to reduce mother-to-infant transmission further.^17^ Other HBV-endemic Asian countries, such as South Korea and China, do not have prevention and treatment programs of comparable scope and coverage.^45,46^ We expect virus-induced ESLD to be well-controlled in Taiwan within 15 years (in 2034). By that time, the vaccinated cohorts will have reached age 50 years, and nonviral risk factors of ELSD (eg, metabolic factors, alcohol-related factors, genetic factors, socioeconomic status, and accessibility to health care) may become more critical.

### Limitations

This study has some limitations. First, the development of ESLD takes many years, and its occurrence increases rapidly with age. This study followed individuals up to age 39 years, but it only described a tiny fraction of the ESLD burden in the entire human lifespan. Second, Taiwan is a viral hepatitis–endemic area, and this study focused on the viral causes of ESLD. Still, other risk factors of ELSD, such as metabolic factors, alcohol-related factors, genetic factors, socioeconomic status, and accessibility of health care, are also important. Unfortunately, these factors were not collected in the databases for the analysis. Third, although HCV infection is a lesser cause of ESLD than HBV in Taiwan and anti-HCV therapy had begun being promoted recently, our study has too limited follow-up time to demonstrate its possible association with the burden of ELSD. Fourth, this study successfully correlated the time trends in the burden of ELSD to the national HBV vaccination and antiviral therapy programs. However, for cohorts born well before the 2 programs started (before 1971), the increased trends for CLD mortality and HCC incidence and the declined trend for HCC mortality remained unaccounted.

## Conclusions

The findings of this cohort suggest that since 1979, both HBV vaccination and antiviral therapy programs were associated with a substantially reduced burden of ELSD in Taiwan. Our successful experience may be used as a model to reach the WHO’s goals of eliminating HBV by 2030.

## Notes

Supplement. **eFigure 1.** Age-Specific Rates of Chronic Liver Disease and Cirrhosis Mortality, Hepatocellular Carcinoma Mortality, and Hepatocellular Carcinoma Incidence by Period for Males in Taiwan **eFigure 2.** Age-Specific Rates of Chronic Liver Disease and Cirrhosis Mortality, Hepatocellular Carcinoma Mortality, and Hepatocellular Carcinoma Incidence by Period for Females in Taiwan **eFigure 3.** Age-Specific Rates of Chronic Liver Disease and Cirrhosis Mortality, Hepatocellular Carcinoma Mortality, and Hepatocellular Carcinoma Incidence by Period in Urban (Metropolis and Cities) Areas of Taiwan **eFigure 4.** Age-Specific Rates of Chronic Liver Disease and Cirrhosis Mortality, Hepatocellular Carcinoma Mortality, and Hepatocellular Carcinoma Incidence by Period in Rural (Towns and Villages) Areas of Taiwan **eFigure 5.** Age-Specific Rates of Chronic Liver Disease and Cirrhosis Mortality, Hepatocellular Carcinoma Mortality, and Hepatocellular Carcinoma Incidence by Cohort for Males in Taiwan **eFigure 6.** Age-Specific Rates of Chronic Liver Disease and Cirrhosis Mortality, Hepatocellular Carcinoma Mortality, and Hepatocellular Carcinoma Incidence by Cohort for Females in Taiwan **eFigure 7.** Age-Specific Rates of Chronic Liver Disease and Cirrhosis Mortality, Hepatocellular Carcinoma Mortality, and Hepatocellular Carcinoma Incidence by Cohort in Urban (Metropolis and Cities) Areas of Taiwan **eFigure 8.** Age-Specific Rates of Chronic Liver Disease and Cirrhosis Mortality, Hepatocellular Carcinoma Mortality, and Hepatocellular Carcinoma Incidence by Cohort in Rural (Towns and Villages) Areas of Taiwan **eFigure 9.** Associations of Age, Period, and Cohort With Chronic Liver Disease and Cirrhosis Mortality, Hepatocellular Carcinoma Mortality, and Hepatocellular Carcinoma Incidence for Males in Taiwan **eFigure 10.** Associations of Age, Period, and Cohort With Chronic Liver Disease and Cirrhosis Mortality, Hepatocellular Carcinoma Mortality, and Hepatocellular Carcinoma Incidence for Females in Taiwan **eFigure 11.** Associations of Age, Period, and Cohort With Chronic Liver Disease and Cirrhosis Mortality, Hepatocellular Carcinoma Mortality, and Hepatocellular Carcinoma Incidence in Urban (Metropolis and Cities) Areas of Taiwan **eFigure 12.** Associations of Age, Period, and Cohort With Chronic Liver Disease and Cirrhosis Mortality, Hepatocellular Carcinoma Mortality, and Hepatocellular Carcinoma Incidence in Rural (Towns and Villages) Areas of Taiwan **eTable 1.** Model Hierarchy and Sequential Statistical Tests for Chronic Liver Disease and Cirrhosis Mortality Rates, Hepatocellular Carcinoma Mortality Rates, and Hepatocellular Carcinoma Incidence Rates Overall and Stratified by Sex **eTable 2.** Model Hierarchy and Sequential Statistical Tests for Chronic Liver Disease and Cirrhosis Mortality Rates, Hepatocellular Carcinoma Mortality Rates, and Hepatocellular Carcinoma Incidence Rates in Patients in Urban (Metropolis and Cities) and Rural (Towns and Villages) Areas **eTable 3.** Secular Trend of the Hepatitis B Virus Infection Rate in All Individuals With Hepatocellular Carcinoma Incident Cases Aged 5 to 39 Years Between 2011 and 2018, Stratified By Birth Cohort Between 1975 to 1979 and 2000 to 2004
