# Supplementary material for: Association of Nationwide Hepatitis B Vaccination and Antiviral Therapy Programs With End-Stage Liver Disease Burden in Taiwan
Source: JAMA Netw Open. 2022 Jul 18;5(7):e2222367. doi: 10.1001/jamanetworkopen.2022.22367 (PMC9295001; doi:10.1001/jamanetworkopen.2022.22367)
Supplement: Supplement. — eFigure 1. Age-Specific Rates of Chronic Liver Disease and Cirrhosis Mortality, Hepatocellular Carcinoma Mortality, and Hepatocellular Carcinoma Incidence by Period for Males in Taiwan eFigure 2. Age-Specific Rates of Chronic Liver Disease and Cirrhosis Mortality, Hepatocellular Carcinoma Mortality, and Hepatocellular Carcinoma Incidence by Period for Females in Taiwan eFigure 3. Age-Specific Rates of Chronic Liver Disease and Cirrhosis Mortality, Hepatocellular Carcinoma Mortality, and Hepatocellular Carcinoma Incidence by Period in Urban (Metropolis and Cities) Areas of Taiwan eFigure 4. Age-Specific Rates of Chronic Liver Disease and Cirrhosis Mortality, Hepatocellular Carcinoma Mortality, and Hepatocellular Carcinoma Incidence by Period in Rural (Towns and Villages) Areas of Taiwan eFigure 5. Age-Specific Rates of Chronic Liver Disease and Cirrhosis Mortality, Hepatocellular Carcinoma Mortality, and Hepatocellular Carcinoma Incidence by Cohort for Males in Taiwan eFigure 6. Age-Specific Rates of Chronic Liver Disease and Cirrhosis Mortality, Hepatocellular Carcinoma Mortality, and Hepatocellular Carcinoma Incidence by Cohort for Females in Taiwan eFigure 7. Age-Specific Rates of Chronic Liver Disease and Cirrhosis Mortality, Hepatocellular Carcinoma Mortality, and Hepatocellular Carcinoma Incidence by Cohort in Urban (Metropolis and Cities) Areas of Taiwan eFigure 8. Age-Specific Rates of Chronic Liver Disease and Cirrhosis Mortality, Hepatocellular Carcinoma Mortality, and Hepatocellular Carcinoma Incidence by Cohort in Rural (Towns and Villages) Areas of Taiwan eFigure 9. Associations of Age, Period, and Cohort With Chronic Liver Disease and Cirrhosis Mortality, Hepatocellular Carcinoma Mortality, and Hepatocellular Carcinoma Incidence for Males in Taiwan eFigure 10. Associations of Age, Period, and Cohort With Chronic Liver Disease and Cirrhosis Mortality, Hepatocellular Carcinoma Mortality, and Hepatocellular Carcinoma Incidence for Females in Taiwan eFigure [file jamanetwopen-e2222367-s001.pdf]

Supplemental Online Content

Chiang CJ, Jhuang JR, Yang YW, et al. Association of nationwide hepatitis B vaccination and antiviral therapy programs with end-stage liver disease burden in Taiwan. *JAMA Netw Open*. 2022;5(7):e2222367. doi:10.1001/jamanetworkopen.2022.22367

eFigure 1. Age-Specific Rates of Chronic Liver Disease and Cirrhosis Mortality, Hepatocellular Carcinoma Mortality, and Hepatocellular Carcinoma Incidence by Period for Males in Taiwan
eFigure 2. Age-Specific Rates of Chronic Liver Disease and Cirrhosis Mortality, Hepatocellular Carcinoma Mortality, and Hepatocellular Carcinoma Incidence by Period for Females in Taiwan
eFigure 3. Age-Specific Rates of Chronic Liver Disease and Cirrhosis Mortality, Hepatocellular Carcinoma Mortality, and Hepatocellular Carcinoma Incidence by Period in Urban (Metropolis and Cities) Areas of Taiwan
eFigure 4. Age-Specific Rates of Chronic Liver Disease and Cirrhosis Mortality, Hepatocellular Carcinoma Mortality, and Hepatocellular Carcinoma Incidence by Period in Rural (Towns and Villages) Areas of Taiwan
eFigure 5. Age-Specific Rates of Chronic Liver Disease and Cirrhosis Mortality, Hepatocellular Carcinoma Mortality, and Hepatocellular Carcinoma Incidence by Cohort for Males in Taiwan
eFigure 6. Age-Specific Rates of Chronic Liver Disease and Cirrhosis Mortality, Hepatocellular Carcinoma Mortality, and Hepatocellular Carcinoma Incidence by Cohort for Females in Taiwan
eFigure 7. Age-Specific Rates of Chronic Liver Disease and Cirrhosis Mortality, Hepatocellular Carcinoma Mortality, and Hepatocellular Carcinoma Incidence by Cohort in Urban (Metropolis and Cities) Areas of Taiwan
eFigure 8. Age-Specific Rates of Chronic Liver Disease and Cirrhosis Mortality, Hepatocellular Carcinoma Mortality, and Hepatocellular Carcinoma Incidence by Cohort in Rural (Towns and Villages) Areas of Taiwan
eFigure 9. Associations of Age, Period, and Cohort With Chronic Liver Disease and Cirrhosis Mortality, Hepatocellular Carcinoma Mortality, and Hepatocellular Carcinoma Incidence for Males in Taiwan
eFigure 10. Associations of Age, Period, and Cohort With Chronic Liver Disease and Cirrhosis Mortality, Hepatocellular Carcinoma Mortality, and Hepatocellular Carcinoma Incidence for Females in Taiwan
eFigure 11. Associations of Age, Period, and Cohort With Chronic Liver Disease and

© 2022 Chiang CJ et al. *JAMA Network Open.*

Cirrhosis Mortality, Hepatocellular Carcinoma Mortality, and Hepatocellular Carcinoma Incidence in Urban (Metropolis and Cities) Areas of Taiwan
eFigure 12. Associations of Age, Period, and Cohort With Chronic Liver Disease and Cirrhosis Mortality, Hepatocellular Carcinoma Mortality, and Hepatocellular Carcinoma Incidence in Rural (Towns and Villages) Areas of Taiwan
eTable 1. Model Hierarchy and Sequential Statistical Tests for Chronic Liver Disease and Cirrhosis Mortality Rates, Hepatocellular Carcinoma Mortality Rates, and Hepatocellular Carcinoma Incidence Rates Overall and Stratified by Sex
eTable 2. Model Hierarchy and Sequential Statistical Tests for Chronic Liver Disease and Cirrhosis Mortality Rates, Hepatocellular Carcinoma Mortality Rates, and Hepatocellular Carcinoma Incidence Rates in Patients in Urban (Metropolis and Cities) and Rural (Towns and Villages) Areas
eTable 3. Secular Trend of the Hepatitis B Virus Infection Rate in All Individuals With Hepatocellular Carcinoma Incident Cases Aged 5 to 39 Years Between 2011 and 2018, Stratified By Birth Cohort Between 1975 to 1979 and 2000 to 2004

This supplemental material has been provided by the authors to give readers additional information about their work.

eFigure 1. Age-Specific Rates of Chronic Liver Disease and Cirrhosis Mortality, Hepatocellular Carcinoma Mortality, and Hepatocellular Carcinoma Incidence by Period for Males in Taiwan

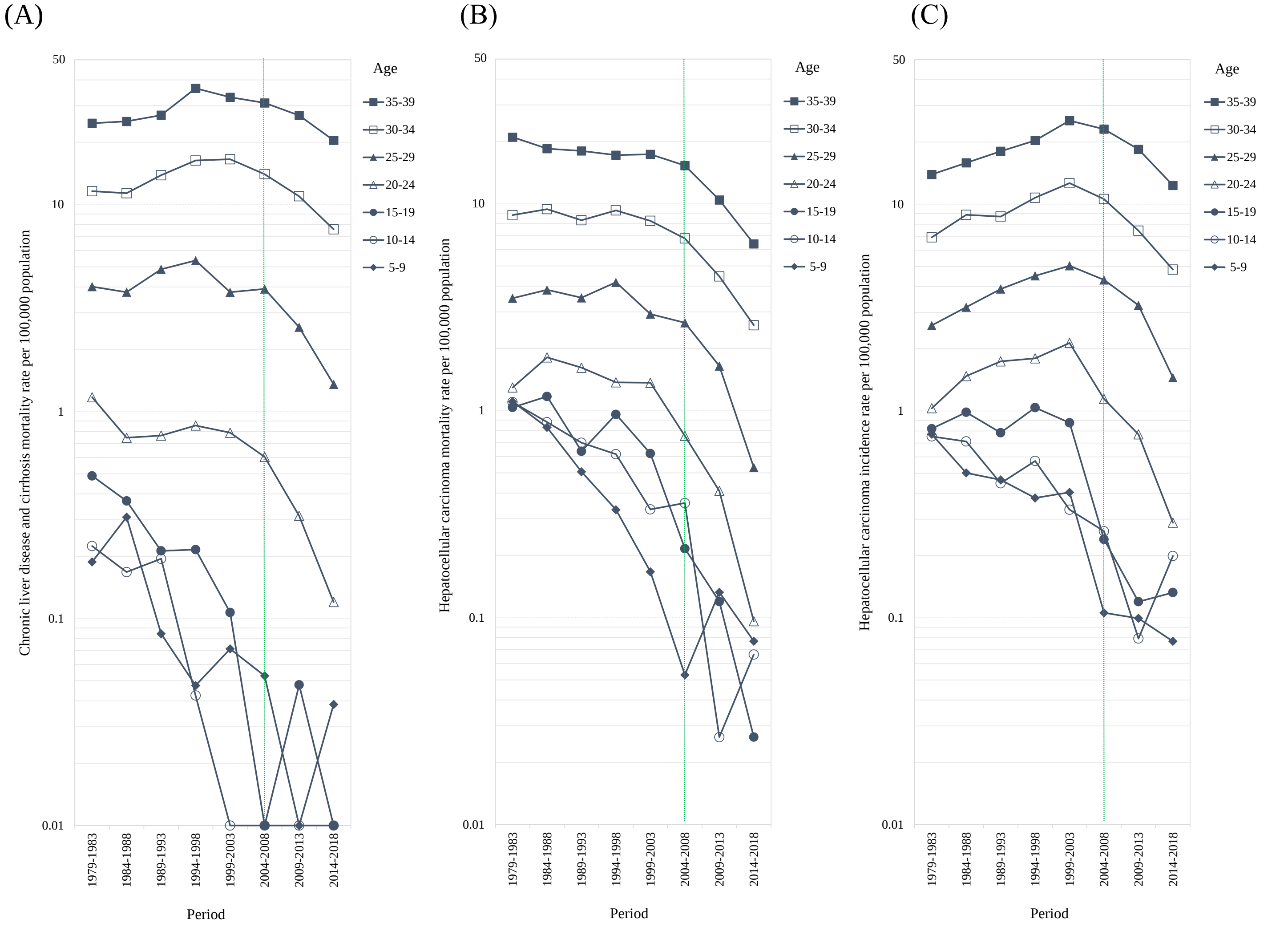

A: Chronic Liver Disease and Cirrhosis Mortality; B: Hepatocellular Carcinoma Mortality; C: Hepatocellular Carcinoma Incidence.

The green dotted line marks the initiation of the antiviral therapy program.

© 2022 Chiang CJ et al. *JAMA Network Open.*

eFigure 2. Age-Specific Rates of Chronic Liver Disease and Cirrhosis Mortality, Hepatocellular Carcinoma Mortality, and Hepatocellular Carcinoma Incidence by Period for Females in Taiwan

(A) (B) (C)

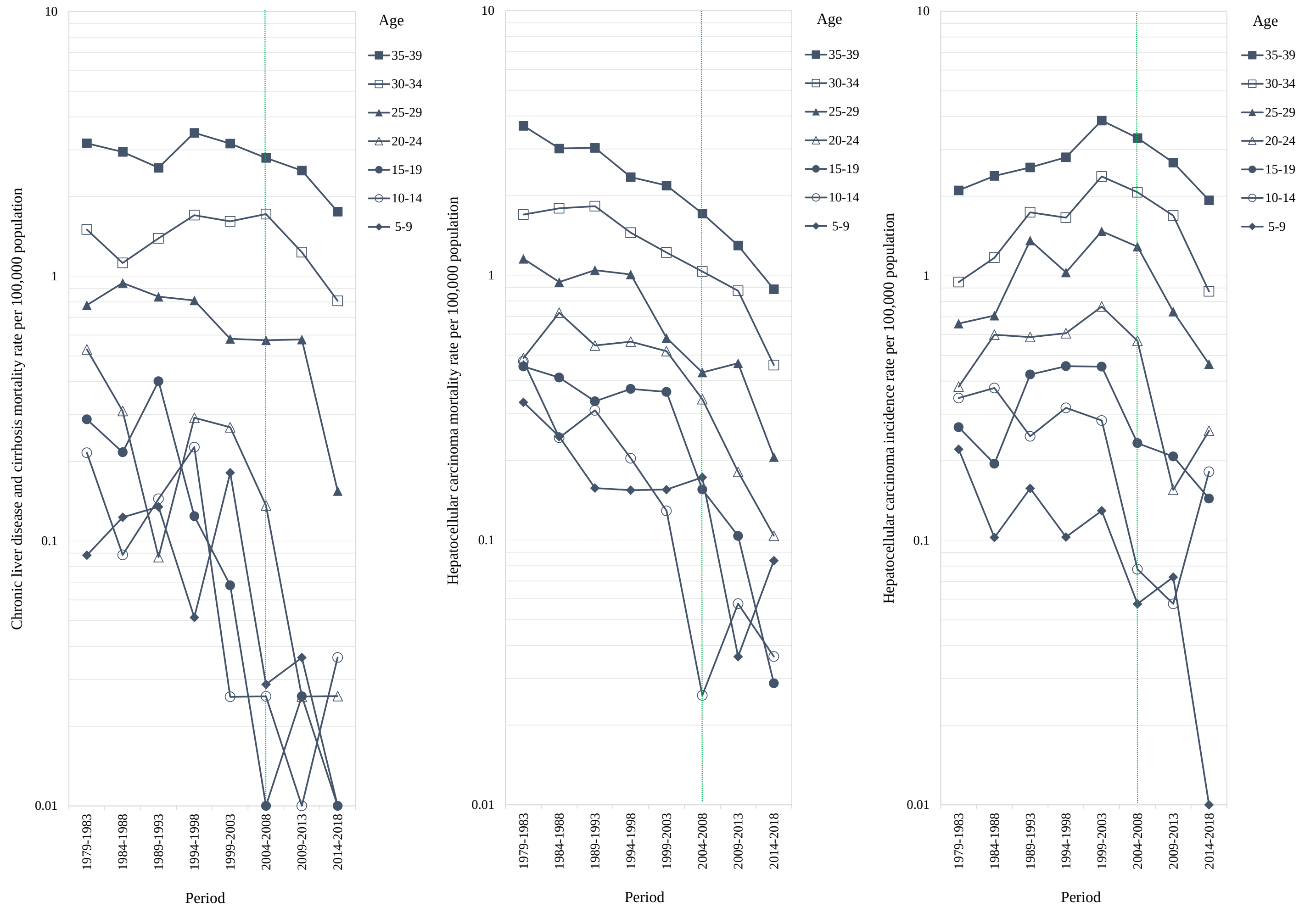

A: Chronic Liver Disease and Cirrhosis Mortality; B: Hepatocellular Carcinoma Mortality; C: Hepatocellular Carcinoma Incidence.

The green dotted line marks the initiation of the antiviral therapy program.

© 2022 Chiang CJ et al. *JAMA Network Open.*

eFigure 3. Age-Specific Rates of Chronic Liver Disease and Cirrhosis Mortality, Hepatocellular Carcinoma Mortality, and Hepatocellular Carcinoma Incidence by Period in Urban (Metropolis and Cities) Areas of Taiwan

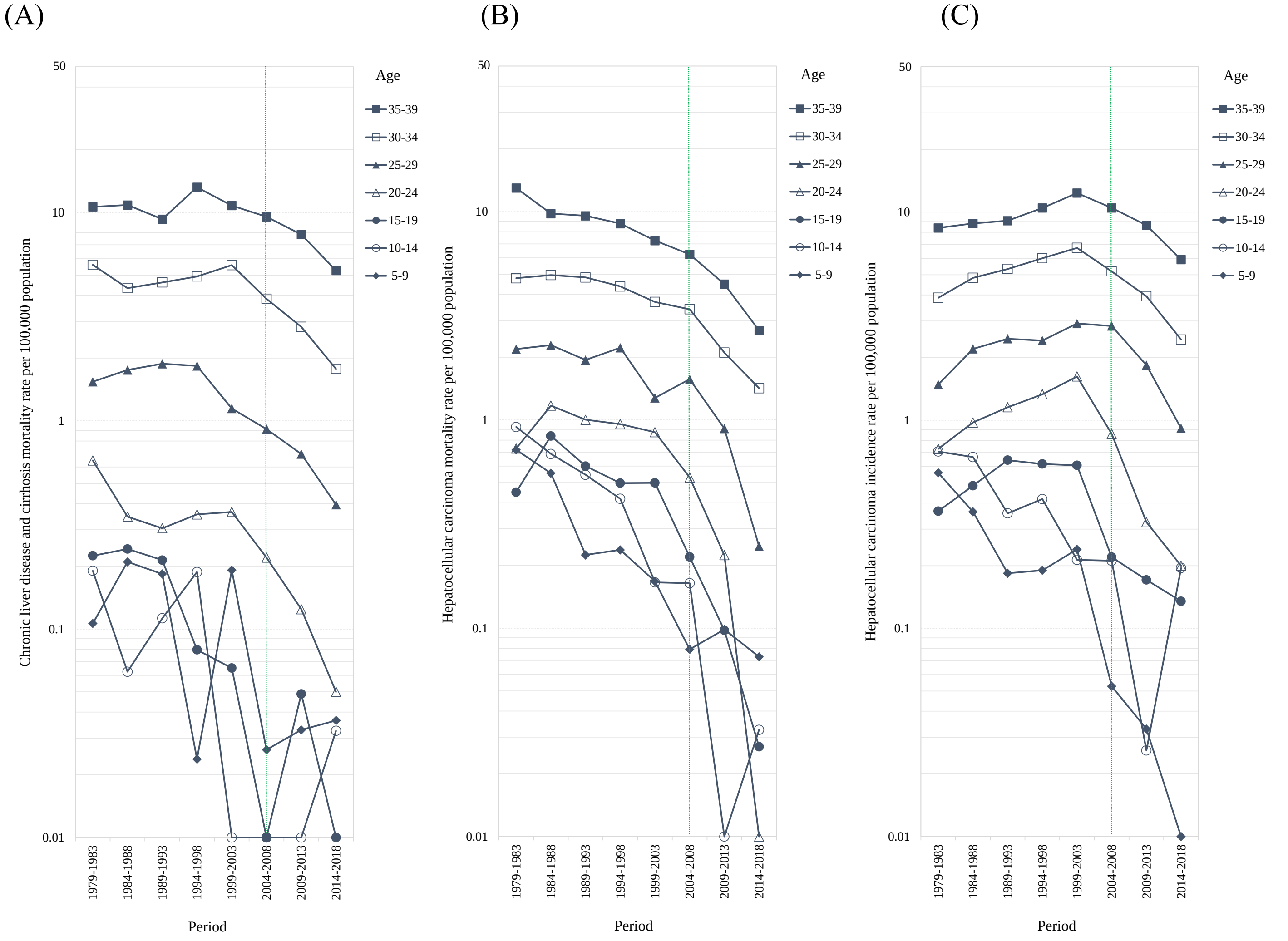

A: Chronic Liver Disease and Cirrhosis Mortality; B: Hepatocellular Carcinoma Mortality; C: Hepatocellular Carcinoma Incidence.

The green dotted line marks the initiation of the antiviral therapy program.

© 2022 Chiang CJ et al. *JAMA Network Open.*

eFigure 4. Age-Specific Rates of Chronic Liver Disease and Cirrhosis Mortality, Hepatocellular Carcinoma Mortality, and Hepatocellular Carcinoma Incidence by Period in Rural (Towns and Villages) Areas of Taiwan

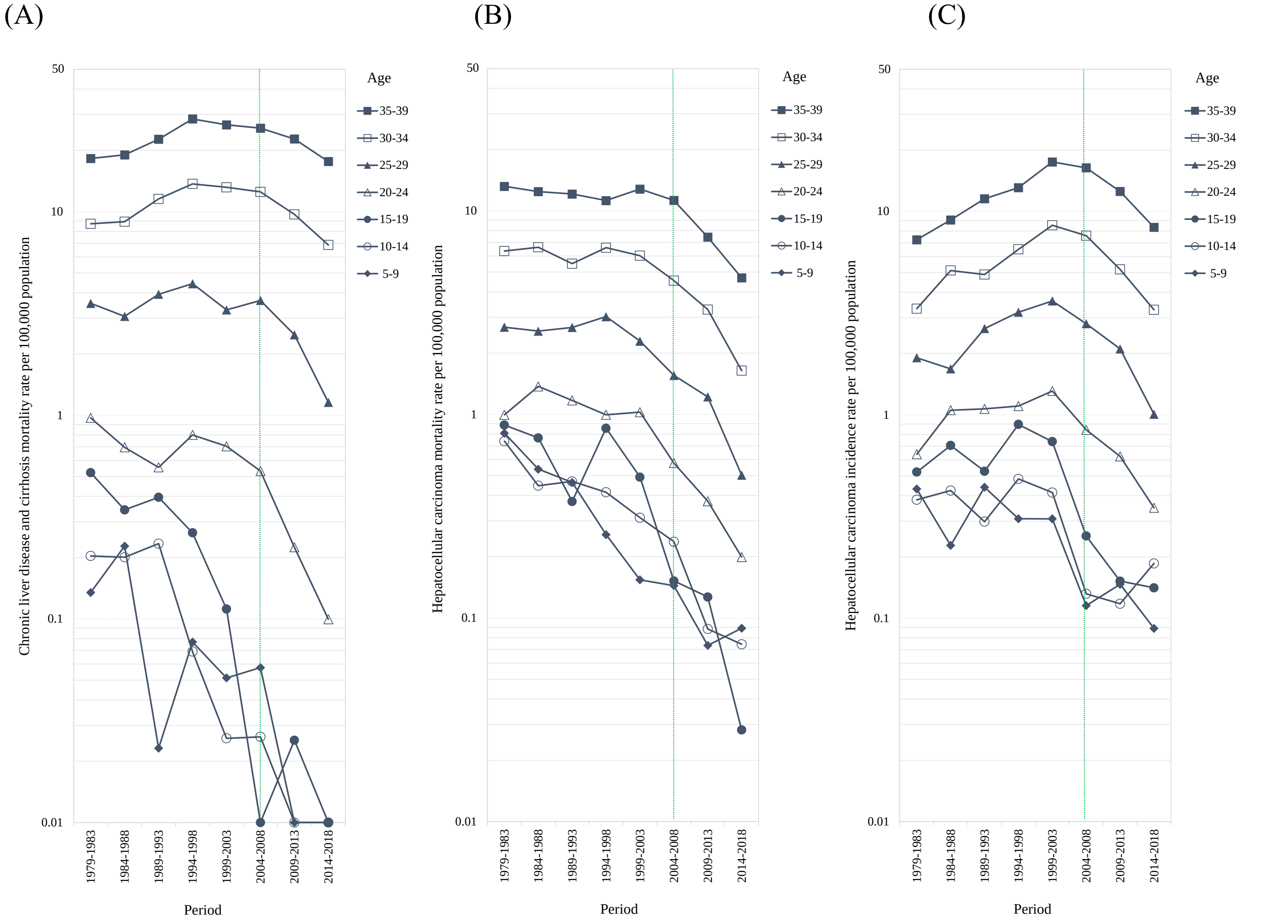

A: Chronic Liver Disease and Cirrhosis Mortality; B: Hepatocellular Carcinoma Mortality; C: Hepatocellular Carcinoma Incidence.

The green dotted line marks the initiation of the antiviral therapy program.

© 2022 Chiang CJ et al. *JAMA Network Open.*

eFigure 5. Age-Specific Rates of Chronic Liver Disease and Cirrhosis Mortality, Hepatocellular Carcinoma Mortality, and Hepatocellular Carcinoma Incidence by Cohort for Males in Taiwan

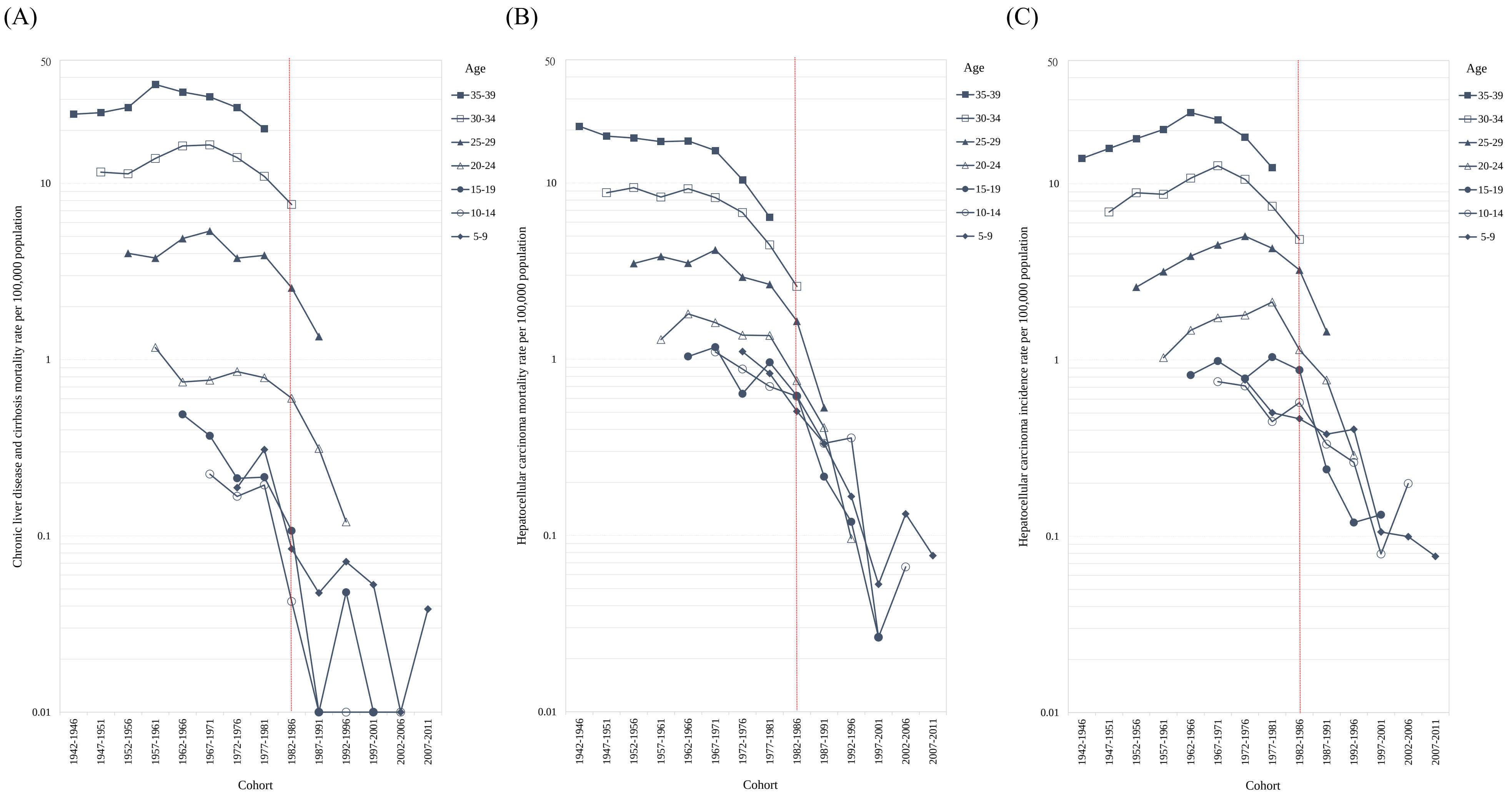

A: Chronic Liver Disease and Cirrhosis Mortality; B: Hepatocellular Carcinoma Mortality; C: Hepatocellular Carcinoma Incidence.

The red dotted line marks the implementation of the hepatitis B vaccination program.

© 2022 Chiang CJ et al. *JAMA Network Open.*

eFigure 6. Age-Specific Rates of Chronic Liver Disease and Cirrhosis Mortality, Hepatocellular Carcinoma Mortality, and Hepatocellular Carcinoma Incidence by Cohort for Females in Taiwan

(A) (B) (C)

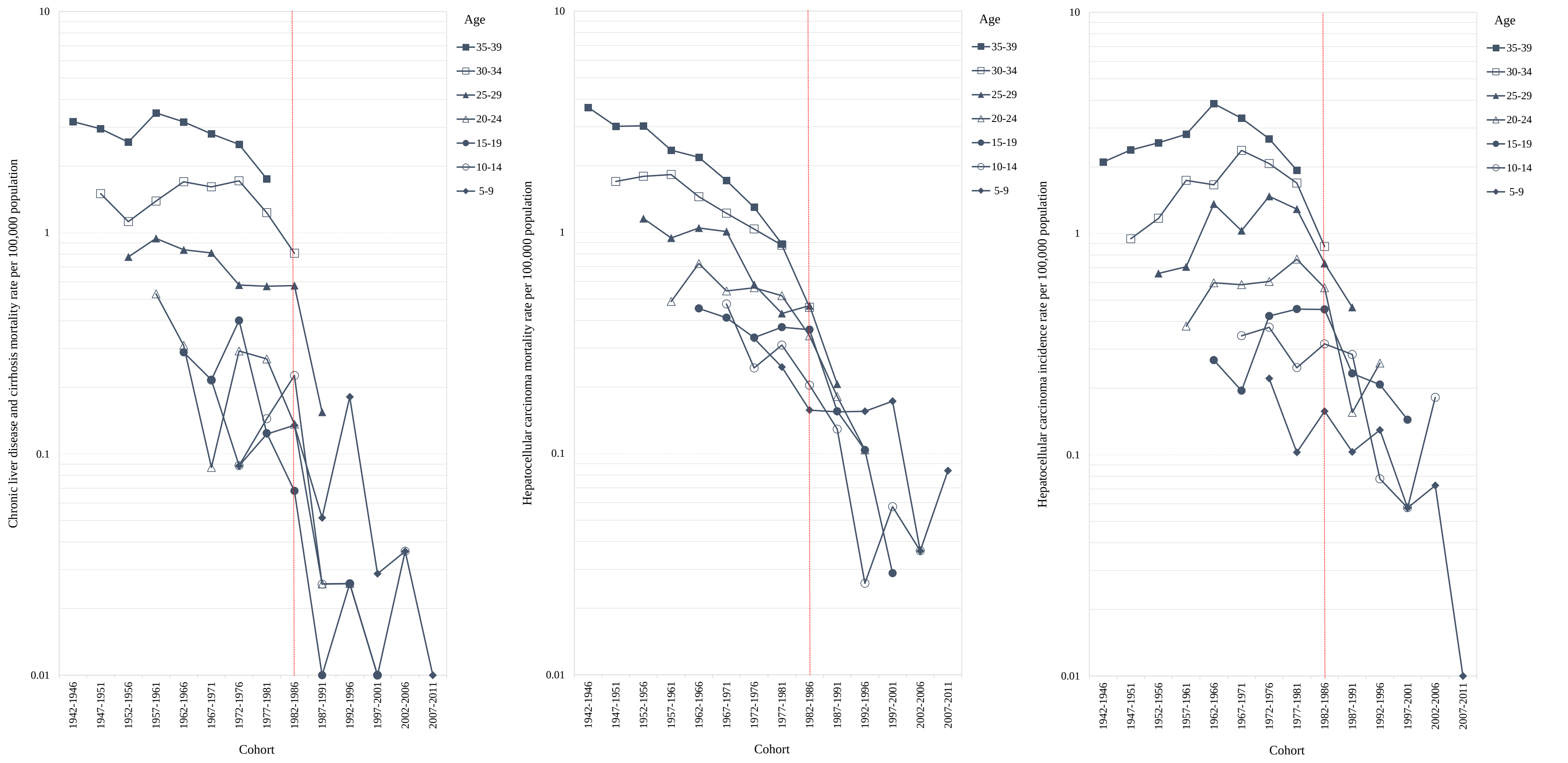

A: Chronic Liver Disease and Cirrhosis Mortality; B: Hepatocellular Carcinoma Mortality; C: Hepatocellular Carcinoma Incidence.

The red dotted line marks the implementation of the hepatitis B vaccination program.

© 2022 Chiang CJ et al. *JAMA Network Open.*

eFigure 7. Age-Specific Rates of Chronic Liver Disease and Cirrhosis Mortality, Hepatocellular Carcinoma Mortality, and Hepatocellular Carcinoma Incidence by Cohort in Urban (Metropolis and Cities) Areas of Taiwan

(A) (B) (C)

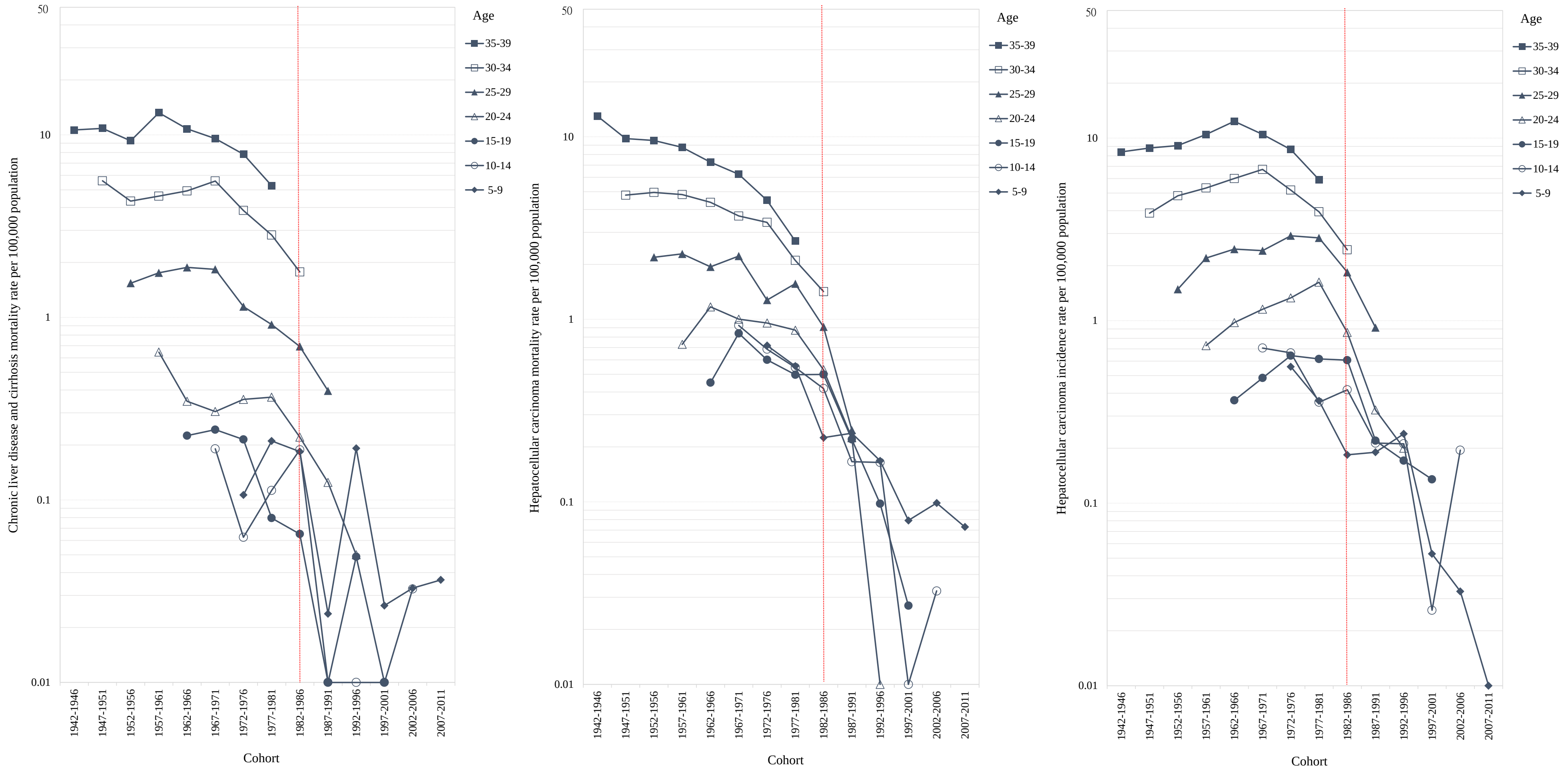

A: Chronic Liver Disease and Cirrhosis Mortality; B: Hepatocellular Carcinoma Mortality; C: Hepatocellular Carcinoma Incidence.

The red dotted line marks the implementation of the hepatitis B vaccination program.

© 2022 Chiang CJ et al. *JAMA Network Open.*

eFigure 8. Age-Specific Rates of Chronic Liver Disease and Cirrhosis Mortality, Hepatocellular Carcinoma Mortality, and Hepatocellular Carcinoma Incidence by Cohort in Rural (Towns and Villages) Areas of Taiwan

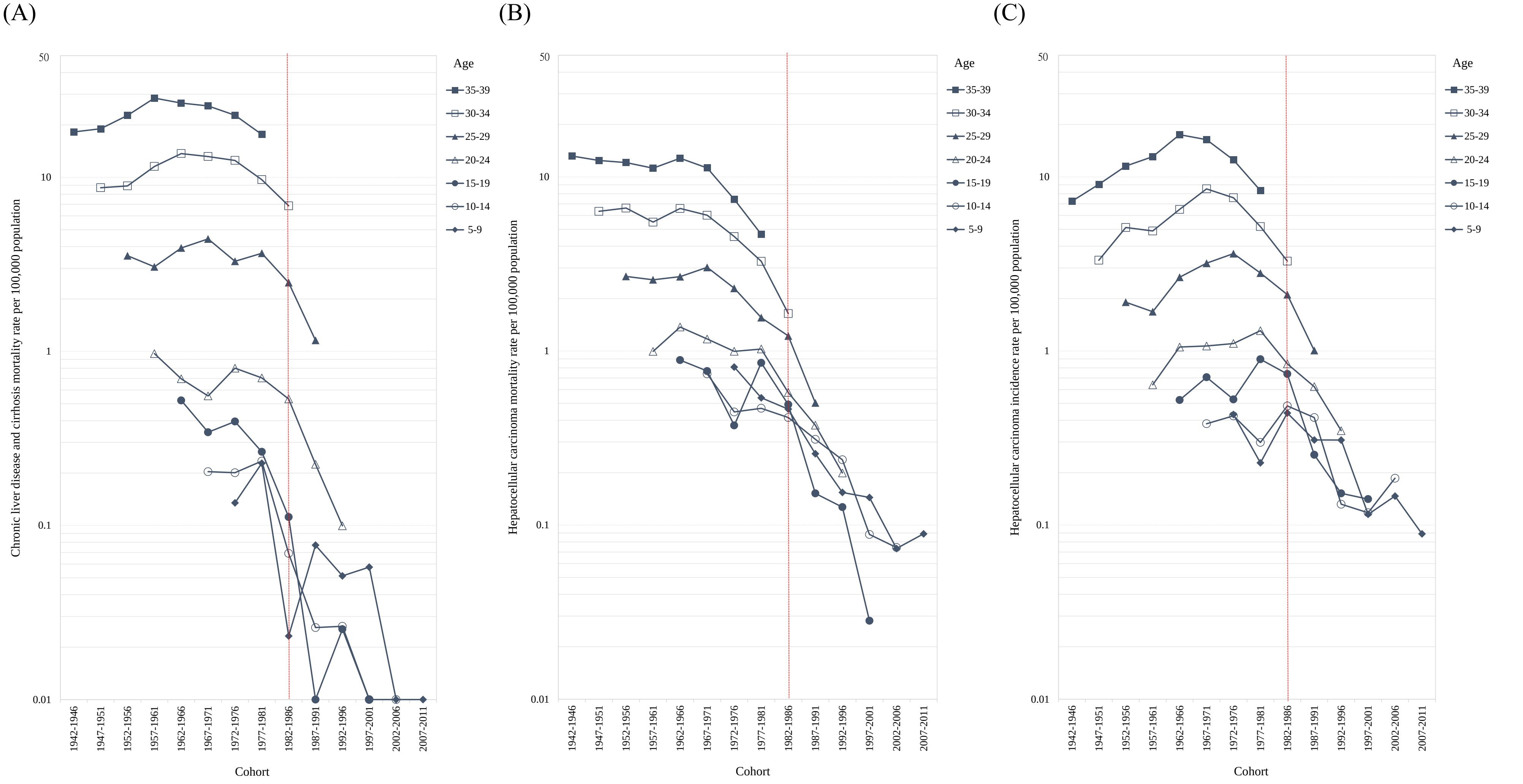

A: Chronic Liver Disease and Cirrhosis Mortality; B: Hepatocellular Carcinoma Mortality; C: Hepatocellular Carcinoma Incidence.

The red dotted line marks the implementation of the hepatitis B vaccination program.

© 2022 Chiang CJ et al. *JAMA Network Open.*

eFigure 9. Associations of Age, Period, and Cohort With Chronic Liver Disease and Cirrhosis Mortality, Hepatocellular Carcinoma Mortality, and Hepatocellular Carcinoma Incidence for Males in Taiwan

A. Association of age, period, and cohort with chronic liver disease and cirrhosis mortality for males in Taiwan.

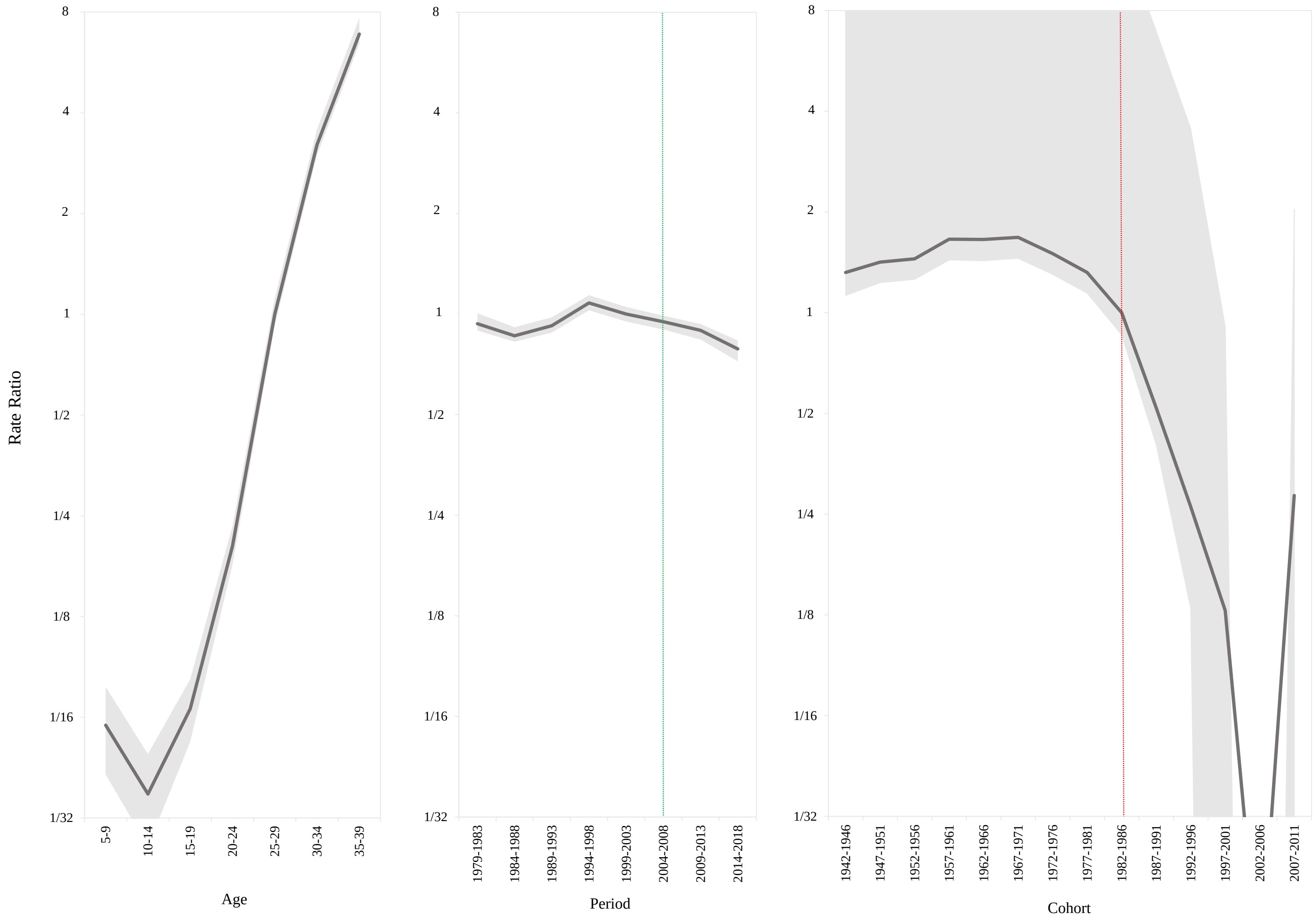

© 2022 Chiang CJ et al. *JAMA Network Open.*

B. Association of age, period, and cohort with hepatocellular carcinoma mortality for males in Taiwan

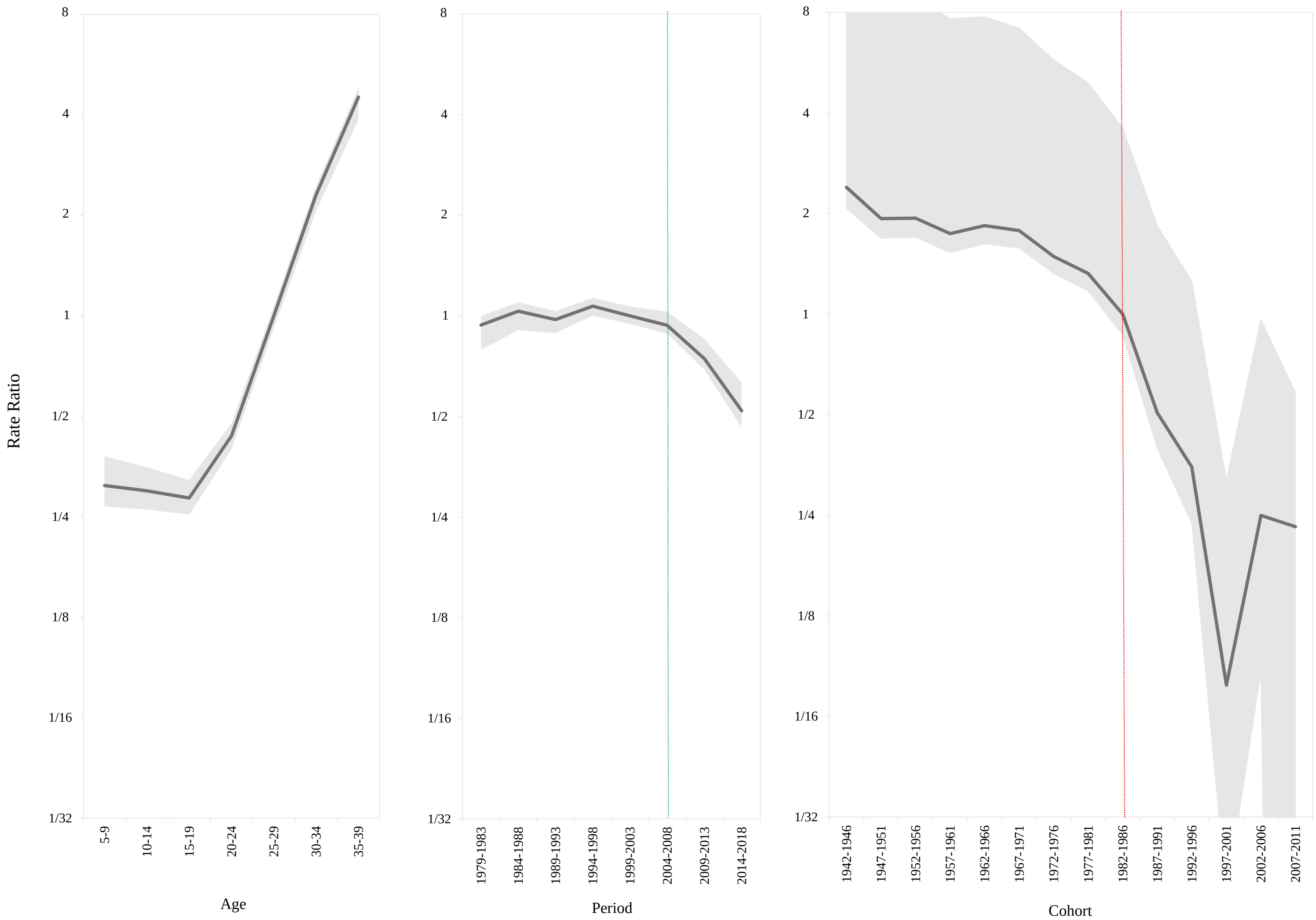

© 2022 Chiang CJ et al. *JAMA Network Open.*

C. Association of age, period, and cohort with hepatocellular carcinoma incidence for males in Taiwan

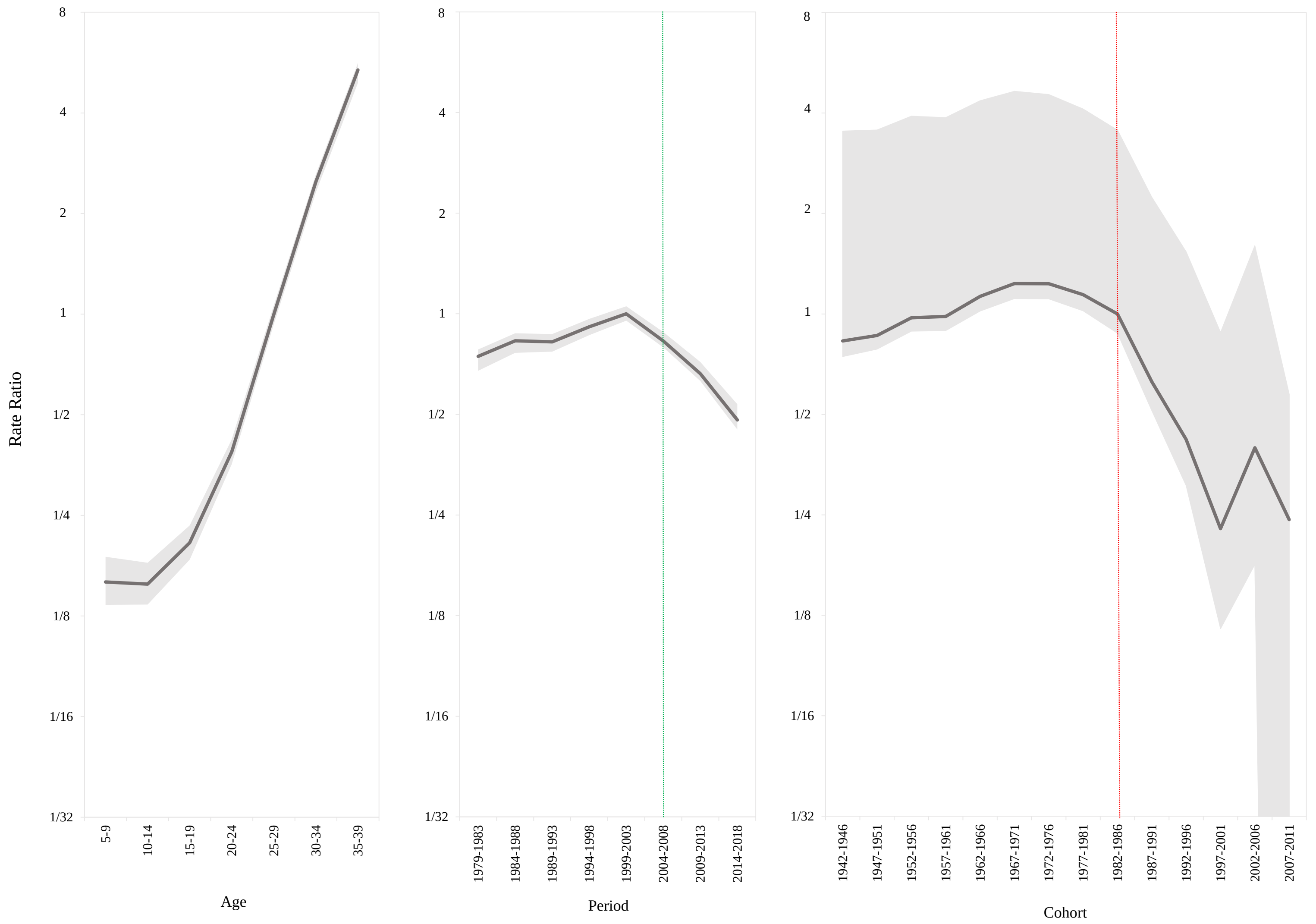

The two dotted lines mark the implementation of the hepatitis B vaccination program (red line) and the initiation of the antiviral therapy program (green line). Shaded areas indicate 95% bootstrapped confidence intervals.

© 2022 Chiang CJ et al. *JAMA Network Open.*

eFigure 10. Associations of Age, Period, and Cohort With Chronic Liver Disease and Cirrhosis Mortality, Hepatocellular Carcinoma Mortality, and Hepatocellular Carcinoma Incidence for Females in Taiwan

A. Association of age, period, and cohort with chronic liver disease and cirrhosis mortality for females in Taiwan

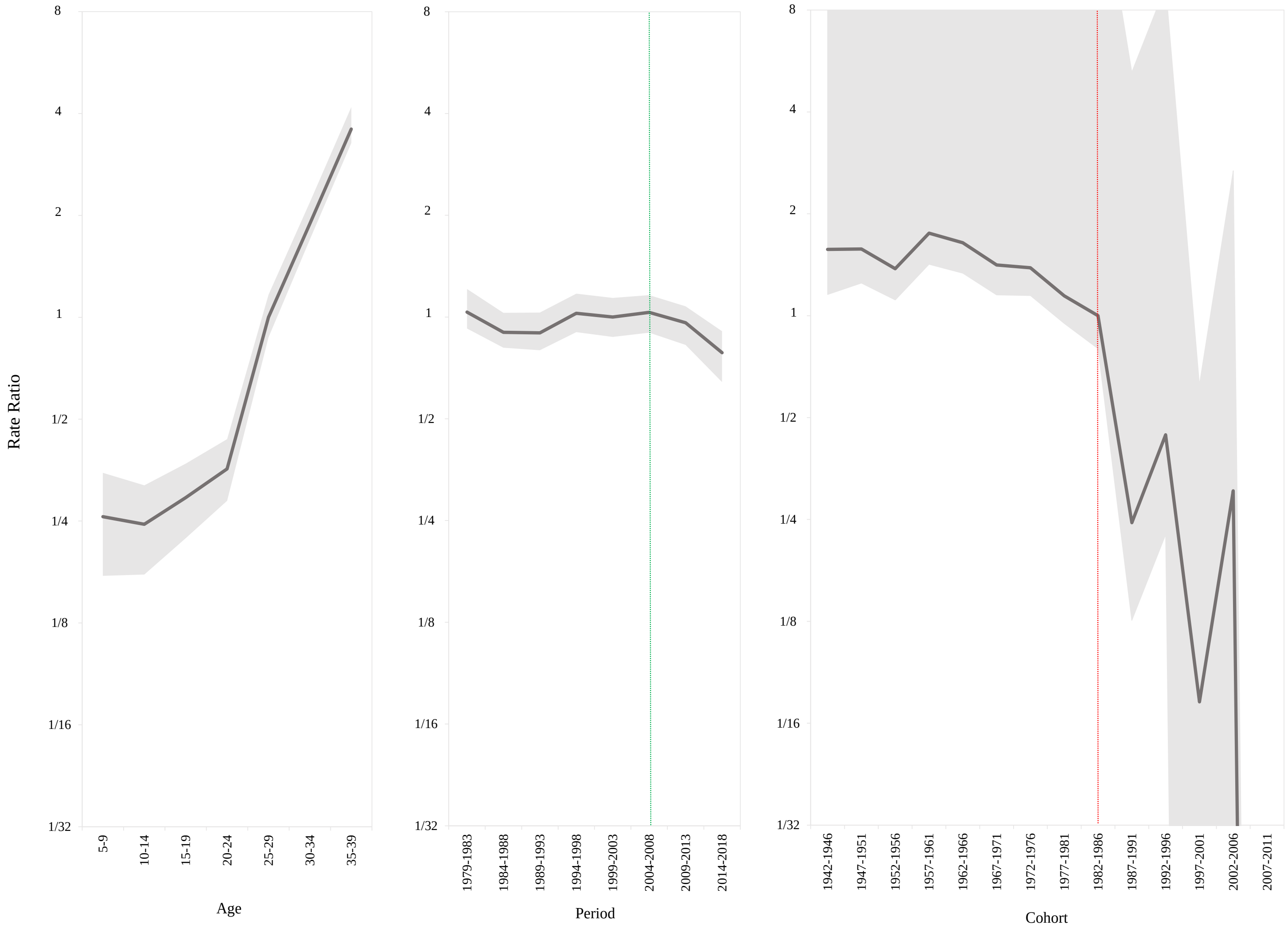

© 2022 Chiang CJ et al. *JAMA Network Open.*

B. Association of age, period, and cohort with hepatocellular carcinoma mortality for females in Taiwan

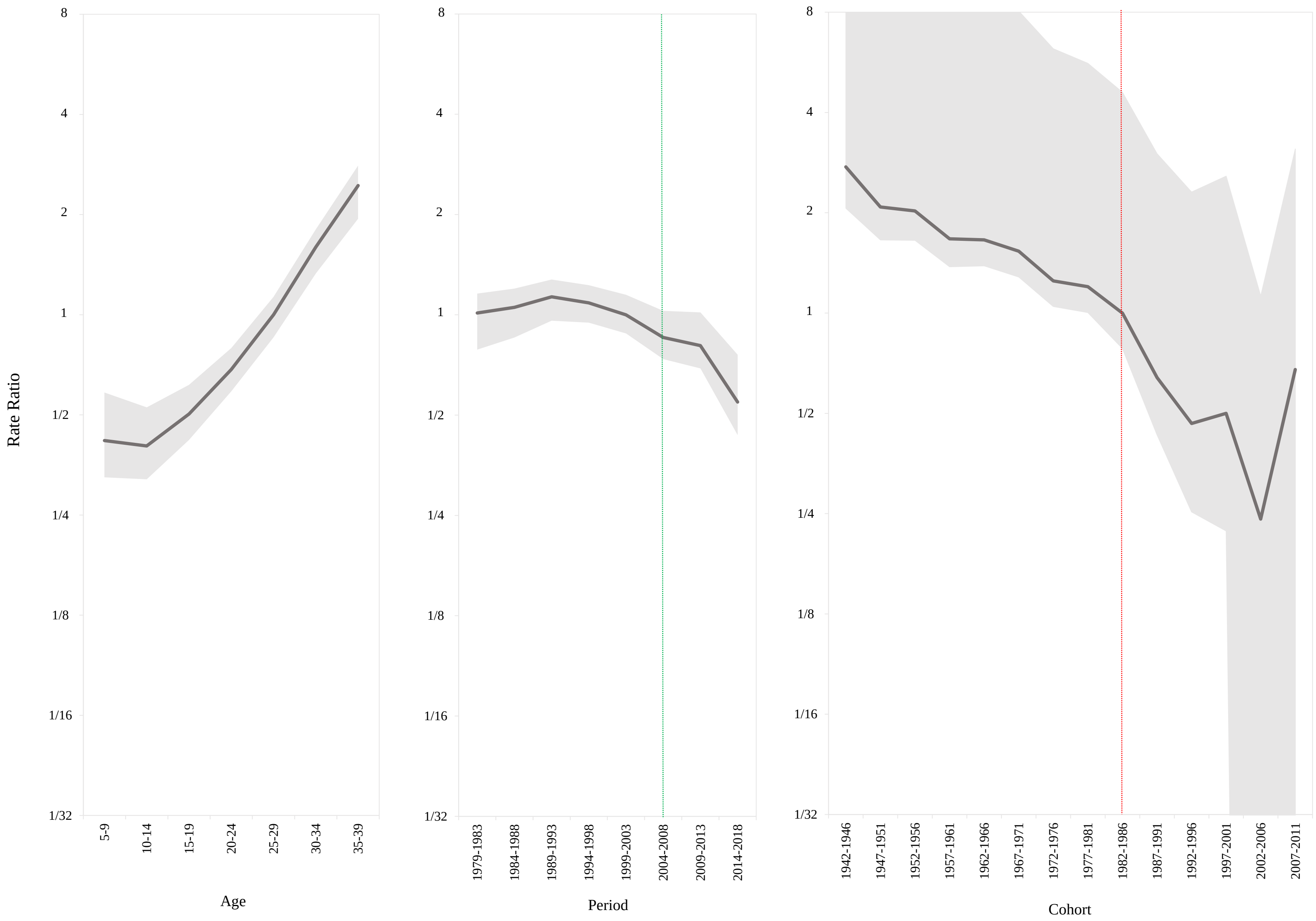

© 2022 Chiang CJ et al. *JAMA Network Open.*

C. Association of age, period, and cohort with hepatocellular carcinoma incidence for females in Taiwan

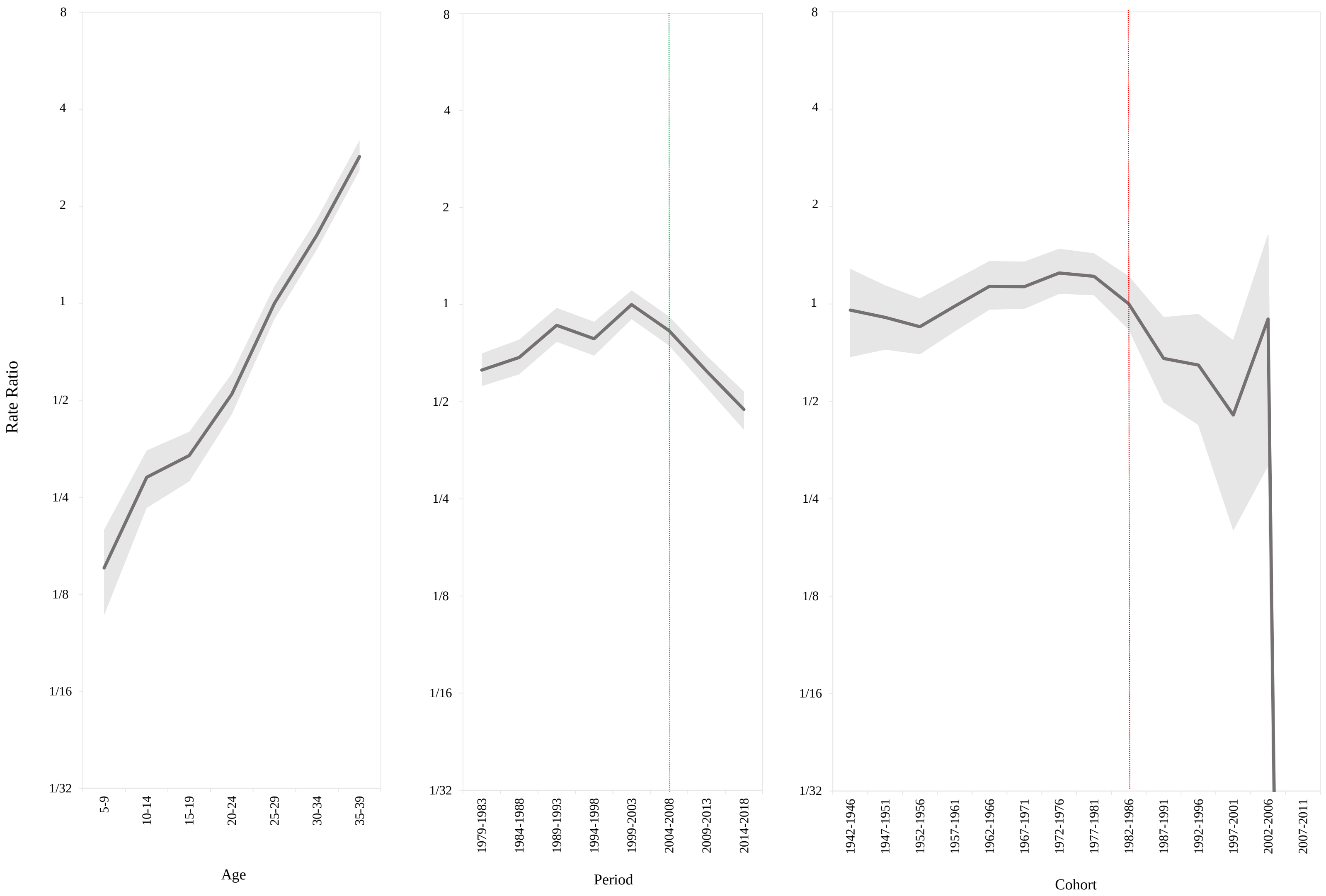

The two dotted lines mark the implementation of the hepatitis B vaccination program (red line) and the initiation of the antiviral therapy program (green line). Shaded areas indicate 95% bootstrapped confidence intervals.

© 2022 Chiang CJ et al. *JAMA Network Open.*

eFigure 11. Associations of Age, Period, and Cohort With Chronic Liver Disease and Cirrhosis Mortality, Hepatocellular Carcinoma Mortality, and Hepatocellular Carcinoma Incidence in Urban (Metropolis and Cities) Areas of Taiwan

A. Association of age, period, and cohort with chronic liver disease and cirrhosis mortality in urban (metropolis and cities) areas of Taiwan

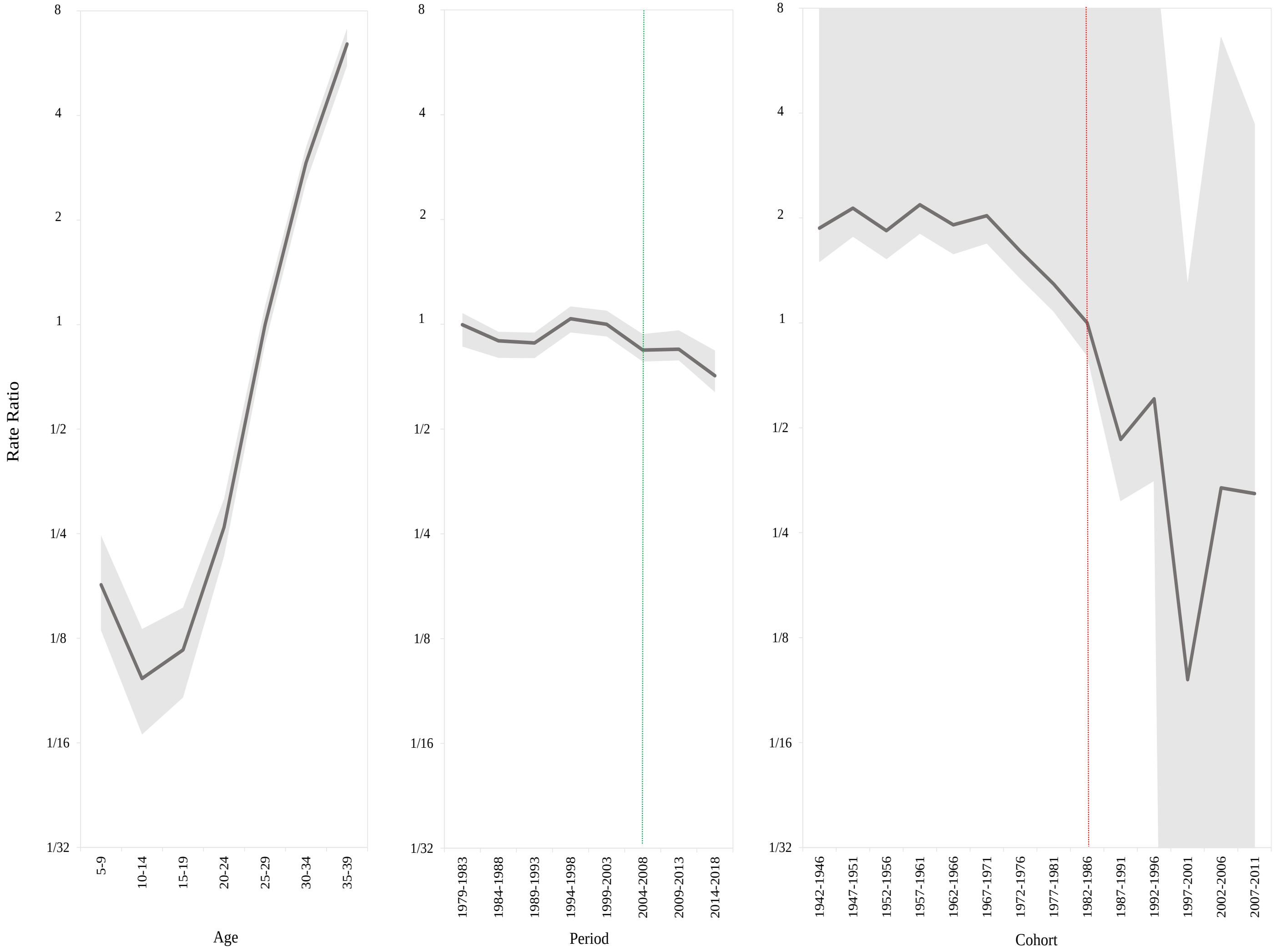

© 2022 Chiang CJ et al. *JAMA Network Open.*

B. Association of age, period, and cohort with hepatocellular carcinoma mortality in urban (metropolis and cities) areas of Taiwan

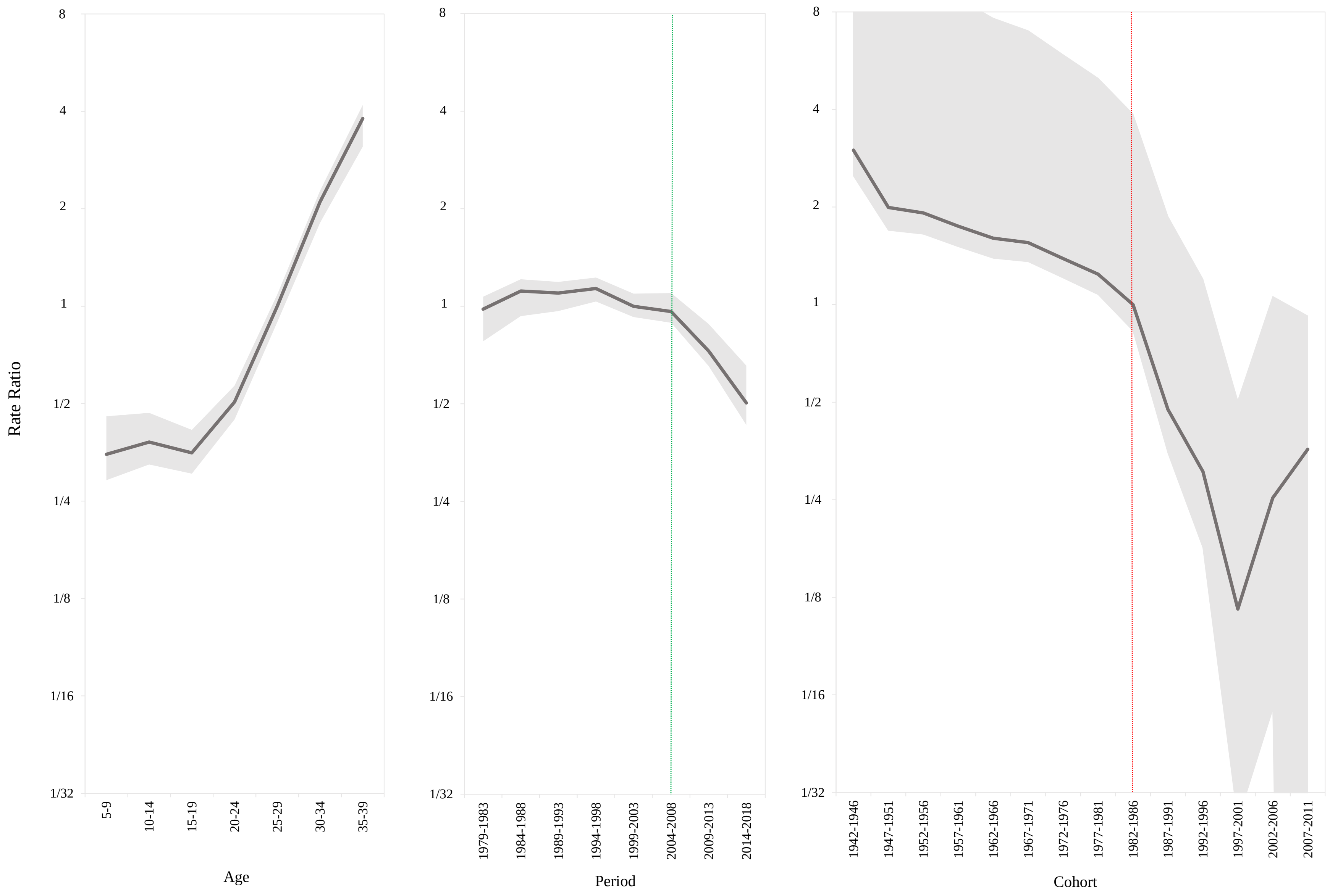

© 2022 Chiang CJ et al. *JAMA Network Open.*

C. Association of age, period, and cohort with hepatocellular carcinoma incidence in urban (metropolis and cities) areas of Taiwan

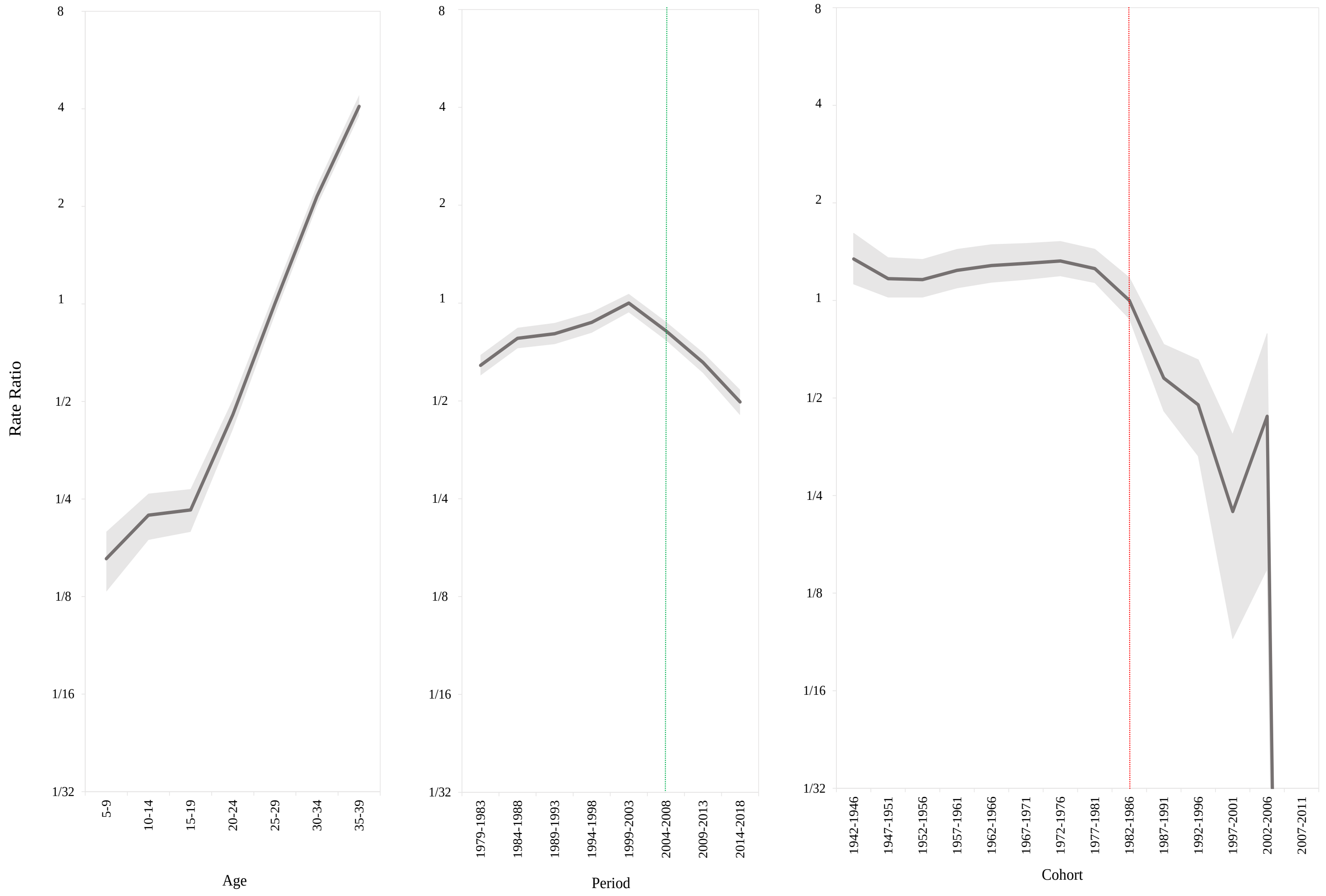

The two dotted lines mark the implementation of the hepatitis B vaccination program (red line) and the initiation of the antiviral therapy program (green line). Shaded areas indicate 95% bootstrapped confidence intervals.

© 2022 Chiang CJ et al. *JAMA Network Open.*

eFigure 12. Associations of Age, Period, and Cohort With Chronic Liver Disease and Cirrhosis Mortality, Hepatocellular Carcinoma Mortality, and Hepatocellular Carcinoma Incidence in Rural (Towns and Villages) Areas of Taiwan

A. Association of age, period, and cohort with chronic liver disease and cirrhosis mortality in rural (towns and villages) areas of Taiwan

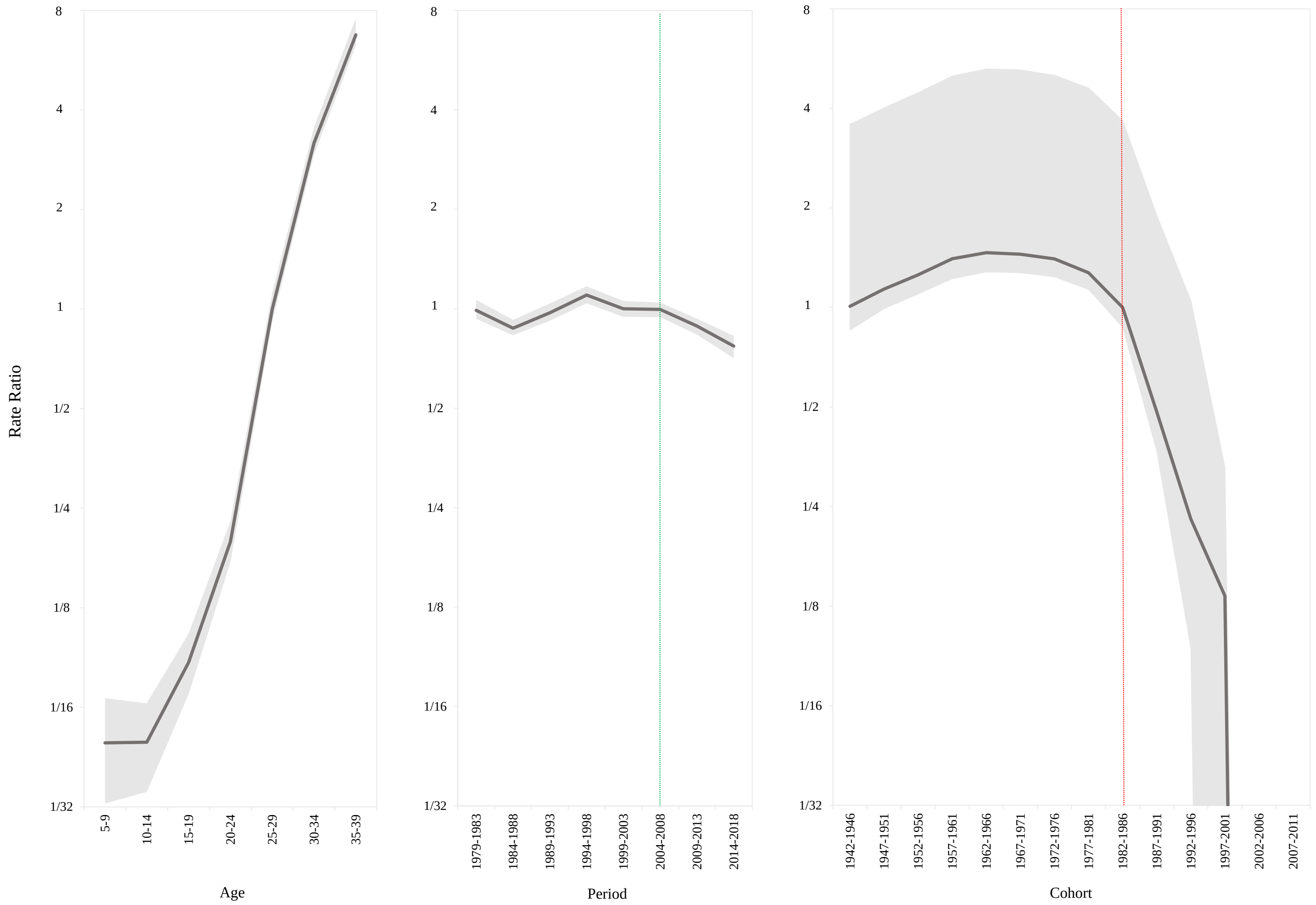

© 2022 Chiang CJ et al. *JAMA Network Open.*

B. Association of age, period, and cohort with hepatocellular carcinoma mortality in rural (towns and villages) areas of Taiwan

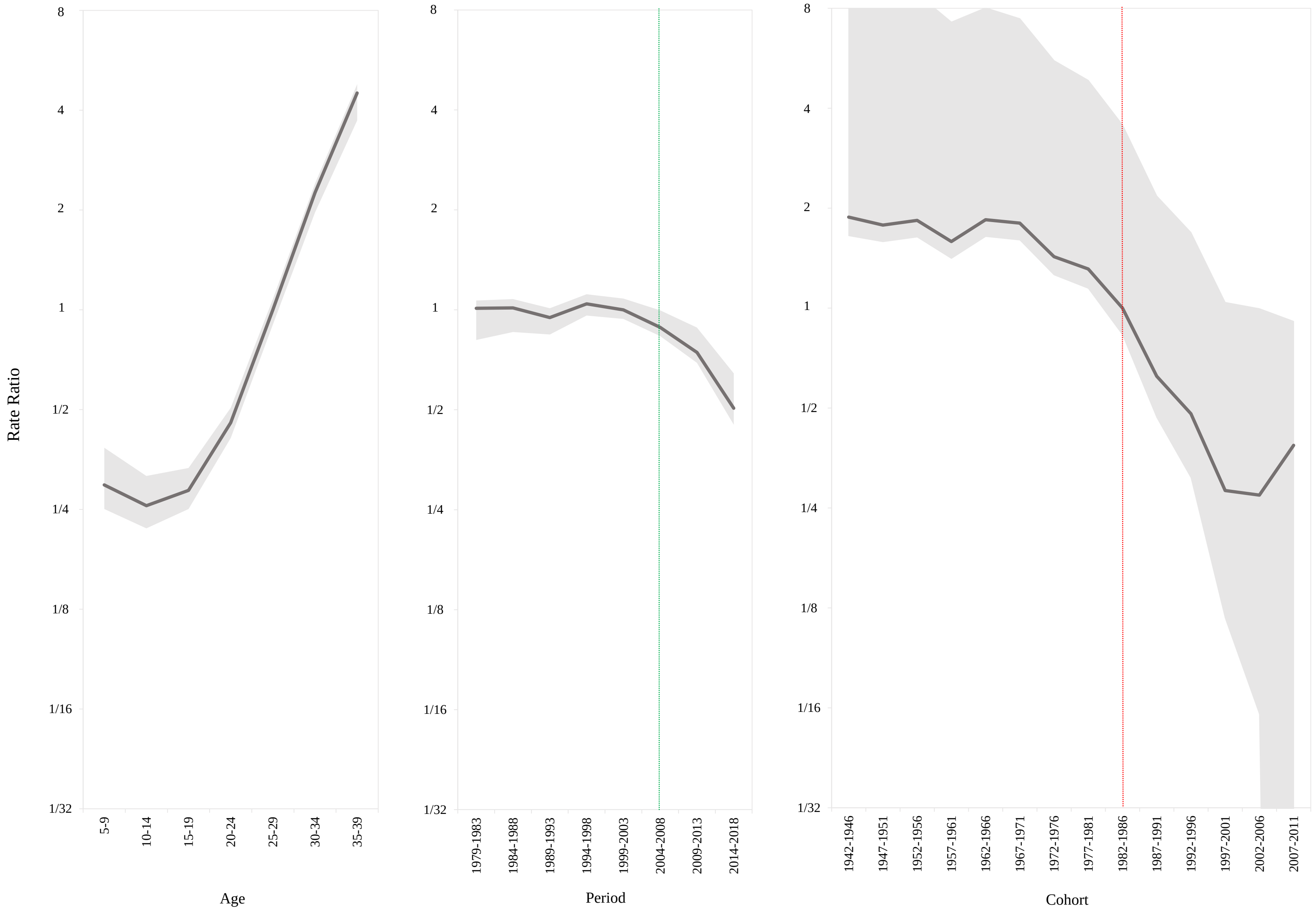

© 2022 Chiang CJ et al. *JAMA Network Open.*

C. Association of age, period, and cohort with hepatocellular carcinoma incidence in rural (towns and villages) areas of Taiwan

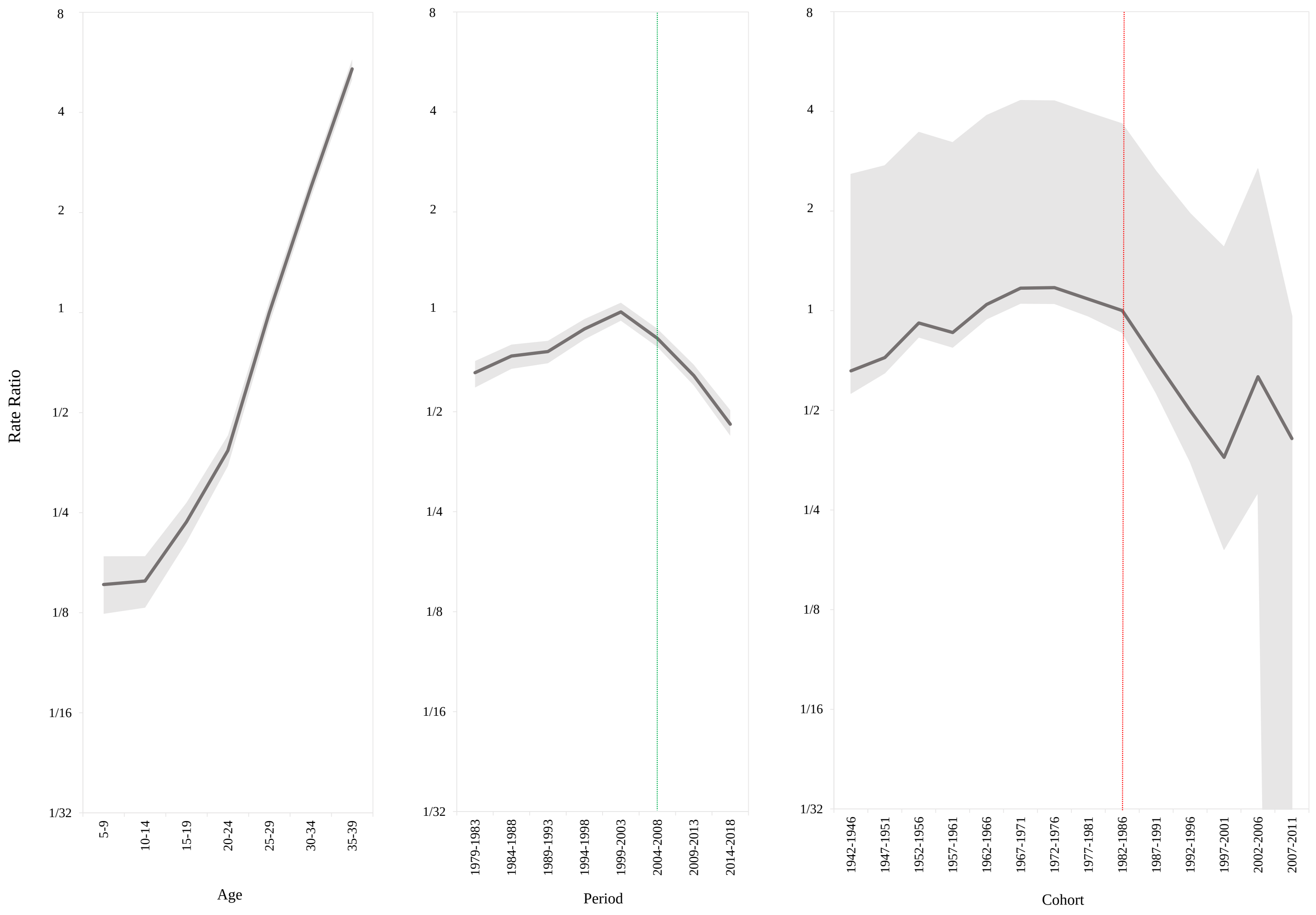

The two dotted lines mark the implementation of the hepatitis B vaccination program (red line) and the initiation of the antiviral therapy program (green line). Shaded areas indicate 95% bootstrapped confidence intervals.

© 2022 Chiang CJ et al. *JAMA Network Open.*

eTable 1. Model Hierarchy and Sequential Statistical Tests for Chronic Liver Disease and Cirrhosis Mortality Rates, Hepatocellular Carcinoma Mortality Rates, and Hepatocellular Carcinoma Incidence Rates Overall and Stratified by Sex

	**Chronic liver disease and cirrhosis mortality**					**Hepatocellular carcinoma mortality**					**Hepatocellular carcinoma incidence**				
Model	*df*	Deviance	Likelihood ratio statistic[a] (*df*[b])		P value (effect tested)	*df*	Deviance	Likelihood ratio statistic[a] (*df*[b])		P value (effect tested)	*df*	Deviance	Likelihood ratio statistic[a] (*df*[b])		P value (effect tested)
**Both sexes combined**															
A	49	1068.18	977.68	(19)	< 0.0001 (P,C)	49	1803.92	1776.67	(19)	< 0.0001 (P,C)	49	1136.95	1091.95	(19)	< 0.0001 (P,C)
P	49	32890.67	32800.32	(19)	< 0.0001 (A,C)	49	14802.63	14775.38	(19)	< 0.0001 (A,C)	49	18387.05	18342.05	(19)	< 0.0001 (A,C)
C	42	20051.79	19961.44	(12)	< 0.0001 (A,P)	42	5972.81	5945.56	(12)	< 0.0001 (A,P)	42	11478.99	11433.99	(12)	< 0.0001 (A,P)
A+P	43	509.87	419.52	(13)	< 0.0001 (C)	43	1083.7	1056.45	(13)	< 0.0001 (C)	43	355.87	310.87	(13)	< 0.0001 (C)
A+C	36	149.04	58.69	(6)	< 0.0001 (P)	36	139.77	112.52	(6)	< 0.0001 (P)	36	302.42	257.42	(6)	< 0.0001 (P)
P+C	36	11098.38	11008.03	(6)	< 0.0001 (A)	36	2447.41	2420.16	(6)	< 0.0001 (A)	36	5543.89	5498.89	(6)	< 0.0001 (A)
A+P+C	30	90.35	Reference		-	30	27.25	Reference		-	30	45.00	Reference		-
**Males**															
A	49	877.41	810.15	(19)	< 0.0001 (P,C)	49	1476.06	1450.15	(19)	< 0.0001 (P,C)	49	962.38	917.31	(19)	< 0.0001 (P,C)
P	49	31188.35	31121.09	(19)	< 0.0001 (A,C)	49	13729.64	13703.73	(19)	< 0.0001 (A,C)	49	17057.17	17012.10	(19)	< 0.0001 (A,C)
C	42	19372.23	19304.97	(12)	< 0.0001 (A,P)	42	5858.89	5832.98	(12)	< 0.0001 (A,P)	42	10641.55	10596.48	(12)	< 0.0001 (A,P)
A+P	43	370.22	302.96	(13)	< 0.0001 (C)	43	896.94	871.03	(13)	< 0.0001 (C)	43	323.42	278.35	(13)	< 0.0001 (C)
A+C	36	118.70	51.44	(6)	< 0.0001 (P)	36	120.93	95.02	(6)	< 0.0001 (P)	36	227.90	182.83	(6)	< 0.0001 (P)
P+C	36	10823.76	10756.50	(6)	< 0.0001 (A)	36	2400.37	2374.46	(6)	< 0.0001 (A)	36	5169.16	5124.09	(6)	< 0.0001 (A)
A+P+C	30	67.26	Reference		-	30	25.91	Reference		-		45.07	Reference		-
**Females**															
A	49	225.62	168.61	(19)	< 0.0001 (P,C)	49	346.00	322.69	(19)	< 0.0001 (P,C)	49	218.77	190.85	(19)	< 0.0001 (P,C)
P	49	2381.12	2324.11	(19)	< 0.0001 (A,C)	49	1437.90	1414.59	(19)	< 0.0001 (A,C)	49	1823.55	1795.63	(19)	< 0.0001 (A,C)
C	42	1215.92	1158.91	(12)	< 0.0001 (A,P)	42	388.66	365.35	(12)	< 0.0001 (A,P)	42	1159.72	1131.80	(12)	< 0.0001 (A,P)
A+P	43	187.78	130.77	(13)	< 0.0001 (C)	43	208.16	184.85	(13)	< 0.0001 (C)	43	85.45	57.53	(13)	< 0.0001 (C)

© 2022 Chiang CJ et al. *JAMA Network Open.*

A+C	36	64.82	7.81	(6)	0.25 (P)	36	40.54	17.23	(6)	< 0.01 (P)	36	94.24	66.32	(6)	< 0.0001 (P)
P+C	36	643.83	586.82	(6)	< 0.0001 (A)	36	165.93	142.62	(6)	< 0.0001 (A)	36	549.21	521.29	(6)	< 0.0001 (A)
A+P+C	30	57.01	Reference		-	30	23.31	Reference		-	30	27.92	Reference		-

Abbreviation: df, degree of freedom; A, age; P, period; C, cohort.

[a] increase in deviance from the age-period-cohort model.

[b] increase in df from the age-period-cohort model.

© 2022 Chiang CJ et al. *JAMA Network Open.*

eTable 2. Model Hierarchy and Sequential Statistical Tests for Chronic Liver Disease and Cirrhosis Mortality Rates, Hepatocellular Carcinoma Mortality Rates, and Hepatocellular Carcinoma Incidence Rates in Patients in Urban (Metropolis and Cities) and Rural (Towns and Villages) Areas

	**Chronic liver disease and cirrhosis mortality**					**Hepatocellular carcinoma mortality**					**Hepatocellular carcinoma incidence**				
Model	*df*	Deviance	Likelihood ratio statistic[*] (*df*[†])		P value	*df*	Deviance	Likelihood ratio statistic[*] (*df*[†])		P value	*df*	Deviance	Likelihood ratio statistic[*] (*df*[†])		P value
**Urban (metropolises/cities) areas**															
A	49	576.30	523.62	(19)	< 0.0001 (P,C)	49	1028.73	982.65	(19)	< 0.0001 (P,C)	49	565.19	513.14	(19)	< 0.0001 (P,C)
P	49	10248.02	10195.34	(19)	< 0.0001 (A,C)	49	6395.67	6349.59	(19)	< 0.0001 (A,C)	49	8209.38	8157.33	(19)	< 0.0001 (A,C)
C	42	5239.49	5186.81	(12)	< 0.0001 (A,P)	42	2124.80	2078.72	(12)	< 0.0001 (A,P)	42	4475.17	4423.12	(12)	< 0.0001 (A,P)
A+P	43	320.59	267.91	(13)	< 0.0001 (C)	43	598.19	552.11	(13)	< 0.0001 (C)	43	228.69	176.64	(13)	< 0.0001 (C)
A+C	36	75.41	22.73	(6)	< 0.001 (P)	36	106.14	60.06	(6)	< 0.0001 (P)	36	169.84	117.79	(6)	< 0.0001 (P)
P+C	36	2722.01	2669.33	(6)	< 0.0001 (A)	36	920.00	873.92	(6)	< 0.0001 (A)	36	2225.76	2173.71	(6)	< 0.0001 (A)
A+P+C	30	52.68	Reference		-	30	46.08	Reference		-	30	52.05	Reference		-
**Rural (towns/villages) areas**															
A	49	641.97	584.47	(19)	< 0.0001 (P,C)	49	879.91	854.08	(19)	< 0.0001 (P,C)	49	674.03	645.99	(19)	< 0.0001 (P,C)
P	49	22869.41	22811.91	(19)	< 0.0001 (A,C)	49	8316.58	8290.75	(19)	< 0.0001 (A,C)	49	9879.78	9851.74	(19)	< 0.0001 (A,C)
C	42	14658.10	14600.60	(12)	< 0.0001 (A,P)	42	3726.33	3700.50	(12)	< 0.0001 (A,P)	42	6718.55	6690.51	(12)	< 0.0001 (A,P)
A+P	43	270.63	213.13	(13)	< 0.0001 (C)	43	502.71	476.88	(13)	< 0.0001 (C)	43	159.41	131.37	(13)	< 0.0001 (C)
A+C	36	103.48	45.98	(6)	< 0.0001 (P)	36	80.57	54.74	(6)	< 0.0001 (P)	36	187.62	159.58	(6)	< 0.0001 (P)
P+C	36	8359.29	8301.79	(6)	< 0.0001 (A)	36	1553.29	1527.46	(6)	< 0.0001 (A)	36	3231.58	3203.54	(6)	< 0.0001 (A)
A+P+C	30	57.50	Reference		-	30	25.83	Reference		-	30	28.04	Reference		-

Abbreviation: df, degree of freedom; A, age; P, period; C, cohort.

[a] increase in deviance from the age-period-cohort model.

[b] increase in df from the age-period-cohort model.

© 2022 Chiang CJ et al. *JAMA Network Open.*

eTable 3. Secular Trend of the Hepatitis B Virus Infection Rate in All Individuals With Hepatocellular Carcinoma Incident Cases Aged 5 to 39 Years Between 2011 and 2018, Stratified By Birth Cohort Between 1975 to 1979 and 2000 to 2004

Birth year	All HCC cases	Cases with			HBV infection rate		
		NBNC infection	HCV infection	HBV infection	%	95% confidence interval[a]	
1975-1979	829	118	20	691	83.4	80.6	85.8
1980-1984	497	75	8	414	83.3	79.7	86.5
1985-1989	100	24	3	73	73.0	63.2	81.4
1990-1994	36	14	0	22	61.1	43.5	76.9
1995-1999	15	7	1	7	46.7	21.3	73.4
2000-2004	9	4	0	5	55.6	21.2	86.3

HCC: hepatocellular carcinoma; NBNC: Non-Hepatitis B Virus-Non-Hepatitis C Virus; HCV: Hepatitis C Virus; HBV: Hepatitis B Virus.

All HCC cases aged 5-39 years were collected from the Taiwan Cancer Registry Long-Form (LF) database between 2011 and 2018. Data on the hepatitis virus infection status of all HCC cases should be reported to the LF database after 2011.

[a] two-sided 95% confidence interval of the HBV infection rate was estimated using the exact binomial method.

© 2022 Chiang CJ et al. *JAMA Network Open.*
